# The Effects of Non-pharmaceutical Interventions on COVID-19 Mortality: A Generalized Synthetic Control Approach Across 169 Countries

**DOI:** 10.3389/fpubh.2022.820642

**Published:** 2022-04-04

**Authors:** Sebastian Mader, Tobias Rüttenauer

**Affiliations:** ^1^Institute of Sociology, University of Bern, Bern, Switzerland; ^2^Nuffield College, University of Oxford, Oxford, United Kingdom

**Keywords:** COVID-19, global public health, health policy, non-pharmaceutical interventions, lockdown, vaccination

## Abstract

**Importance:**

Governments have introduced non-pharmaceutical interventions (NPIs) in response to the pandemic outbreak of Coronavirus disease (COVID-19). While NPIs aim at preventing fatalities related to COVID-19, the previous literature on their efficacy has focused on infections and on data of the first half of 2020. Still, findings of early NPI studies may be subject to underreporting and missing timeliness of reporting of cases. Moreover, the low variation in treatment timing during the first wave makes identification of robust treatment effects difficult.

**Objective:**

We enhance the literature on the effectiveness of NPIs with respect to the period, the number of countries, and the analytical approach.

**Design, Setting, and Participants:**

To circumvent problems of reporting and treatment variation, we analyse data on daily confirmed COVID-19-related deaths per capita from Our World in Data, and on 10 different NPIs from the Oxford COVID-19 Government Response Tracker (OxCGRT) for 169 countries from 1st July 2020 to 1st September 2021. To identify the causal effects of introducing NPIs on COVID-19-related fatalities, we apply the generalized synthetic control (GSC) method to each NPI, while controlling for the remaining NPIs, weather conditions, vaccinations, and NPI-residualized COVID-19 cases. This mitigates the influence of selection into treatment and allows to model flexible post-treatment trajectories.

**Results:**

We do not find substantial and consistent COVID-19-related fatality-reducing effects of any NPI under investigation. We see a tentative change in the trend of COVID-19-related deaths around 30 days after strict stay-at-home rules and to a slighter extent after workplace closings have been implemented. As a proof of concept, our model is able to identify a fatality-reducing effect of COVID-19 vaccinations. Furthermore, our results are robust with respect to various crucial sensitivity checks.

**Conclusion:**

Our results demonstrate that many implemented NPIs may not have exerted a significant COVID-19-related fatality-reducing effect. However, NPIs might have contributed to mitigate COVID-19-related fatalities by preventing exponential growth in deaths. Moreover, vaccinations were effective in reducing COVID-19-related deaths.

## Highlights

- We enhance the existing literature on the effect of non-pharmaceutical interventions (NPIs) on COVID-19-related deaths with respect to the period (1), the number of countries (2), and the analytical approach (3).
- (1) We analyze data on COVID-19-related deaths per capita from Our World in Data, and on 10 different NPIs from the Oxford COVID-19 Government Response Tracker of 169 countries from 1st July 2020 to 1st September 2021 to attenuate identification problems stemming from low variation in treatment (timing) and data quality.
- (2) Compared to other studies, we use data of more countries (*n* = 169) covering 98% of the world population.
- (3) We apply the Generalized Synthetic Control (GSC) method to mitigate the influence of selection into treatment and to model flexible post-treatment trajectories.
- Applying the GSC method, we do not find substantial and consistent COVID-19-related fatality-reducing effects of any NPI under investigation.
- Using the same approach, we find however a significant and substantial fatality-reducing effect of COVID-19 vaccinations.
- The results of our study provide further guidance to judge the effectiveness of NPIs for reducing COVID-19-related fatalities.

## Introduction

Governments have introduced non-pharmaceutical interventions (NPIs) in response to the pandemic outbreak of Coronavirus disease (COVID-19) since early 2020 (see Supplementary Figures 1, 2). NPIs include traditional epidemiological instruments like public information campaigns, testing strategies, as well as contact tracing, and the isolation of infected and vulnerable people. Moreover, obligations to wear a face mask have been introduced. A large share of countries has implemented various lockdown-related NPIs. These include the closure of schools, workplaces and public transport, restrictions on public events and gatherings, and more stringent measures like stay-at-home requirements, as well as restrictions on domestic and international movement (1).

While the potential benefits of stringent NPIs are straightforward (mitigate COVID-19-related fatalities and maltreatment of other diseases due to health system overload), they are likely to be jeopardized by potentially severe or even “prohibitive” (2) economic, social, and public health-related negative externalities associated with the implementation of these NPIs (3–8). Hence, evaluation of the proportionality of NPIs heavily hinges on consistent empirical evidence of their efficacy in the ongoing pandemic (9).

Ever since, a valuable body of empirical NPI-studies has emerged to judge the efficacy of various measures (see Supplementary Table 1 in the Supplementary Material Part E for an overview). However, several potential limitations of the previous literature impede the judgment of the NPIs' efficacy. First, NPIs ultimately aim at avoiding fatalities related to COVID-19, e.g., by preventing the health system to collapse. However, earlier studies on their efficacy have primarily focused on infections. Only ten of the 37 studies listed in Supplementary Table 1 investigate the effect of NPIs on COVID-19-related deaths (10–19). Although studies on infections provide important insights on how NPIs affect the disease dynamics, they cannot adequately evaluate the main aim of avoiding fatalities. Furthermore, underreporting of infections may be more pronounced than for fatalities. Hence, it is favorable to use data on fatalities (20). Second, former investigations based on data of the first half of 2020 may be subject to underreporting and missing timeliness of reporting during the first viral outbreak (14, 17). Third, the identification of robust treatment effects of NPIs is difficult based on the first wave because of the low variation in treatment type and treatment timing across countries in early 2020 (17, 21) (see Supplementary Figures 3–15).

Against this backdrop and given the heterogeneity in data used, methods applied, and NPIs, time spans and countries/territories analyzed, the results of early NPI studies vary considerably. Early causal analyses on daily COVID-19-related fatalities accounting for population size and the temporal delay of treatment impacts find substantial mitigating effects for the initial “lockdown” (12, 14), school closure (10, 13, 18, 19), workplace closure (18, 19), cancellation of public events (18), stay-at-home orders (16), travel restrictions (15), and mask obligations (11, 15). However, all these studies are based on data of the first wave. Only one study (17) investigates fatalities after the first half of 2020 (August 2020 to January 2021). Based on a hierarchical Bayesian transmission model applied to 7 countries/114 subnational entities, the study (17) identifies substantial mitigating effects for school closure, workplace closure, and firm restrictions on gatherings (see Supplementary Table 1).

We enhance the existing literature on the effect of NPIs on COVID-19-related deaths with respect to the period, the number of countries, and the analytical method. First, we analyse data starting from the second half of 2020 (1st July 2020 to 1st September 2021) to circumvent problems of reporting and treatment variation. Variation in treatment timing is sufficiently high between countries in this time span (Supplementary Figures 3–12). Second, compared to other studies (17), we use data of more countries (*N* = 169) covering 98% of the world population. Third, to estimate the average treatment effect on the treated (ATT), we apply the Generalized Synthetic Control (GSC) method (22). The GSC approach is a combination of the synthetic control approach (23–25) and traditional difference-in-differences (DiD) methods (26). This attenuates the influence of selection into treatment while modeling flexible post-treatment trajectories.

## Materials and Methods

### Data

We combine different data sources on the country level (*N* = 169 countries). First, we derive daily information on confirmed COVID-19 deaths per capita from *Our World in Data* (27). Second, we add daily information on various NPIs and governmental stringency from the Oxford COVID-19 Government Response Tracker (OxCGRT) (1). Our main outcome measure is the daily number of newly confirmed deaths attributed to COVID-19 per million inhabitants. We coded all days as missing on which a country reported a negative number of deaths, which happened in a few instances when countries corrected earlier numbers.

From OxCGRT (1) we use the following 10 NPIs: school closing, workplace closing, closure of public transport, stay at home rules, restriction of internal movement, restrictions on international travel, protection of the elderly, testing policy, contact tracing, and mask obligations. OxCGRT codes a country's stringency in each of these domains with different categories. For our main analyses we recoded all NPIs as binary indicators, taking the value one (implemented) if a country is in the highest available category of each measure on a given day, and zero otherwise. Supplementary Figures 3–12 depict the stringency sequences for each NPI.

### Analytical Strategy

Unobserved differences between countries and temporal shocks likely influence the number of COVID-19 related cases and deaths. Hence, a natural choice would be a difference- in-differences (DiD) like estimator (28), solely relying on within country and period variances for identification (28, 29). However, conventional within-estimators face several methodological challenges when aiming at identifying the impact of NPIs on COVID-19 related fatalities, such as selection on pre-treatment trends or treatment effect heterogeneity (26, 30–32).

We tackle this problem by applying a Generalized Synthetic Control (GSC) method (22). The intuitive idea behind the conventional Synthetic Control method is to construct a synthetic control unit for a single treated unit by re-weighting observations from the pool of control-units (23–25). Among other characteristics, the re-weighting is based on the pre-treatment pathway of the outcome. The method thus compares the treatment unit to a weighted control-pool which, on average, has a similar pre-treatment outcome trajectory. Moreover, by predicting the counterfactual outcome for the treated observations, heterogeneous treatment effects over time are flexibly identified.

The traditional Synthetic Control approach applies only to the case of one treated unit. Yet, the GSC method (22) provides a framework which generalizes the synthetic control method to multiple treated units by using factor augmented models. We start with the general model


(1) $$\begin{array}{r} Y_{it}=\delta_{it}D_{it}+X_{it}\beta +L_{it}+\varepsilon_{it}, \end{array}$$

where *Y*_*it*_ is the dependent variable (fatalities per capita), ***X***_*it*_ are time-varying controls, and ε_*it*_ the idiosyncratic error. *D*_*it*_ is a binary treatment indicator, and δ_*it*_ denotes the treatment effect for each unit and time-period, with $\bar{\delta }$_*it*_ being the average treatment effect on the treated (ATT). ***L***_*it*_ subsumes unobserved factors approximating the outcome trend. The original GSC method based on interactive fixed effects models directly estimates ***L*** = **Λ*F***, with **Λ** being an *N* × *r* matrix of unknown factor loadings (unit-specific intercepts), and ***F*** an *r* × *T* matrix of unobserved common factors (time-varying coefficients) (22). This however requires the number of factors r to be specified correctly, for instance by using cross-validation methods.

In contrast to interactive fixed effects models, the matrix completion method (33) does not estimate **Λ** and ***F***, but directly estimates $\hat{L}$ based on nuclear norm regularization. Intuitively, $\hat{L}$ is derived by minimizing the sum of squared differences between *Y*_*it*_ and ***L***_*it*_ (plus non-regularized unit and time fixed effects) based on pre-treatment observations while adding a penalty term λ **||*L*||**—similar to the Least Absolute Shrinkage and Selection Operator (LASSO) estimator. The matrix completion method in GSC is used to estimate the ATT in the following way (22, 34). First, $\hat{\beta }$ and $\hat{L}$ are estimated based on the pool of control units, where λ is determined by cross-validation (33). Here, we use 20 possible λ values and 10-fold cross-validation. Second, the counterfactual outcome for the treated units is estimated by ${\hat{Y}}_{it}(D\;=\;0)\;=X_{it}\hat{\beta }+{\hat{L}}_{it}$. Third, based on the predicted counterfactual outcome, we can then estimate the ATT $\hat{\delta }$_*it*_ = *Y*_*it*_(*D* = 1) – Ŷ_*it*_(*D* = 0). For inferential statistics, we provide confidence intervals based on non-parametric bootstraps of 1,000 runs clustered at the country level (see below). For estimation, we use the R package *gsynth* v.1.1.9 (22).

One limitation of GSC in our case is that the method is designed for settings of staggered treatment adoption. Countries can, however, transition from treatment (having an NPI implemented) to control (relaxing the NPI), and going back to treatment (see Supplement B for treatment sequences). Thus, we divide our data into country-period splits, where a country is treated as a new unit with each transition from treatment to control. A single country can, for instance, act as treated unit in early periods and as control unit in later periods. This also ensures that we do not conflate our temporal treatment effect of implementation with relaxations of NPIs.

To analyse the effectiveness of each single NPI_i_ with *i* ϵ {1*, ...*, 10}, we simultaneously control for the stringency index in all other NPIs: Index_*j*_, *j* ≠*i*. We follow the OxCGRT methodology for normalized sub-index scores by defining Index_*j*_ = 100$\frac{v_{j}-0.5\left(\max\left\{F_{j}-f_{j}\right\}\right)}{N_{vj}}$, where *v*_*j*_ is the current category of the measure, *N*_*vj*_ the maximum category (highest stringency), *f*_*j*_ a binary flag indicating whether a measure is geographically targeted (0) or general (1), and *F*_*j*_ indicates if measure *j* has a flag or not (e.g., international travel restrictions are always general).

We control for the cumulative number of vaccinations per capita (27), as vaccinations likely affect both—the implementation of NPIs and the number of fatalities. Because COVID-19 transmissions (35–37) as well as COVID-19 related morbidity (38) might be correlated with seasonal weather conditions, we also control for the monthly average temperature and temperature squared, cloud cover, specific humidity, and precipitation. These data were derived from the ERA5 reanalysis data based on Copernicus Climate observations (39).

Moreover, we control for temporal lags of residualized COVID-19 cases. Besides the COVID-19 deaths, also the cases are likely to impose a strong effect on the likelihood of implementing NPIs. However, naively controlling for cases would lead to an overcontrol-bias, as NPIs reduce deaths not only directly but also through a reduction in infections. We thus follow a regression-with-residuals approach (40, 41), making COVID-19 cases orthogonal to past NPI implementations. More specifically, we first regress cases on various time-lags of NPIs within countries and time-periods: $Cases_{it}=\overset{10}{\underset{k=1}{\sum }}\overset{35}{\underset{l=7}{\sum }}NPI_{it-l}^{k}+\alpha_{i}\;+\xi_{t}\;$for all K NPIs and L temporal lags, where α_*i*_ and ξ_*t*_ are country and time fixed effects. Subsequently, we derive the residuals of this first-stage regression. This leaves us with the residualized COVID-19 cases which are independent of NPI implementations within the past 7–35 days. In our main analysis, we control for the residualized 7-day backwards rolling average of residualized COVID-19 cases at five different temporal lags: *t* – 7, *t* – 14, *t* – 21, *t* – 28, and *t* – 35, including second and third order polynomials. The high number of lags in the first and second stage intends to relax a-priori assumptions on the temporal dependency between COVID-19 cases, deaths, and NPIs. Controlling for the residualized number of COVID-19 cases notably reduces the pre-treatment differences in control and treatment group (see Supplementary Figure 16 for results without controlling for residualized cases).

## Results

**Figure 1:**
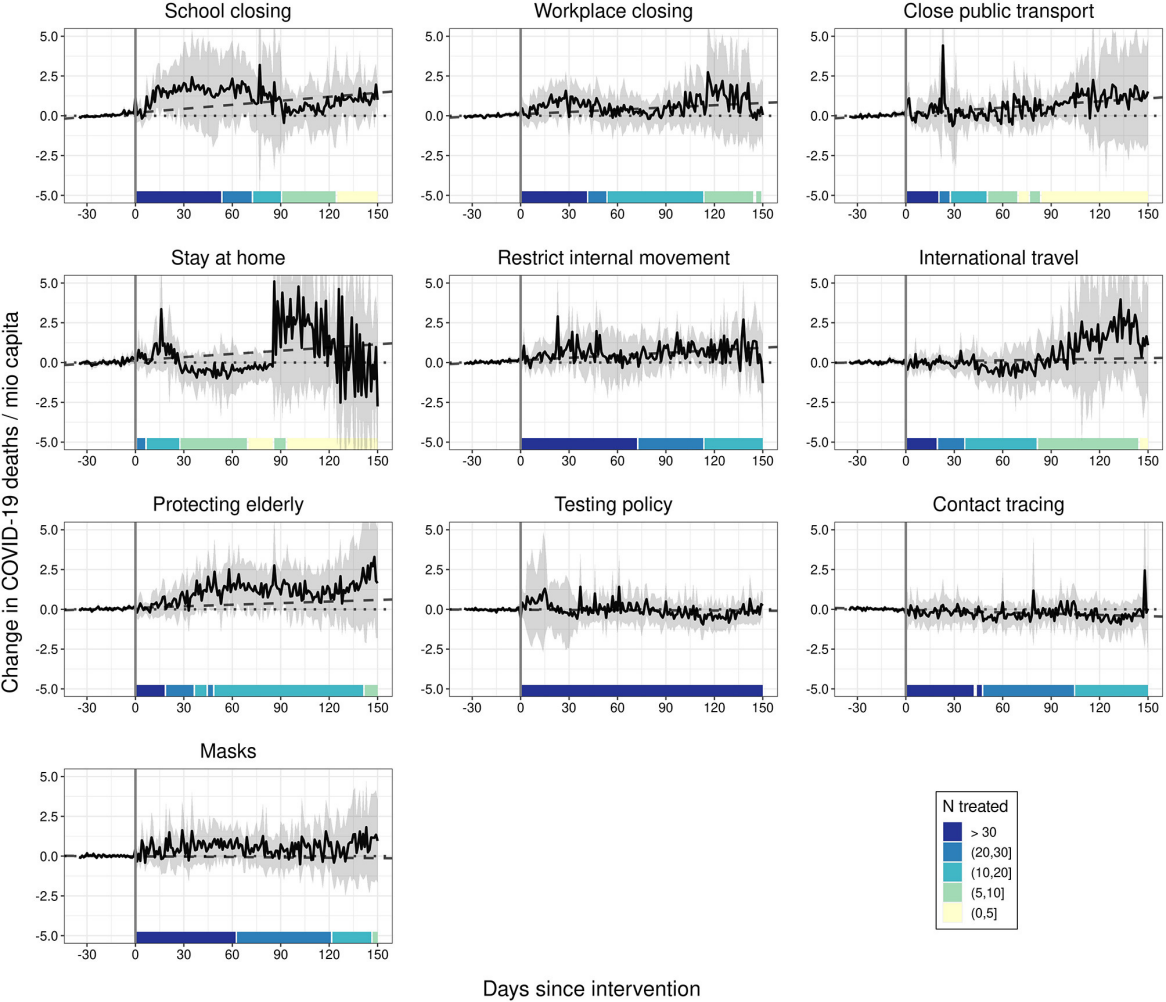
The effect of NPIs on COVID-19 deaths. Generalized synthetic control estimator based on daily data. Black solid lines represent the average treatment effects on the treated (ATTs). Ribbons represent 95% non-parametric confidence intervals based on 1,000 bootstrap runs. Dotted lines are the null lines. Dashed lines represent linear predictions based on the 35 days before the intervention. Controls: 9 remaining NPIs as stringency index, temperature, temperature^2^, cloud cover, precipitation, humidity, total vaccinations, 7-day backwards rolling average of NPI-residualized COVID-19 cases at t – 7, t – 14, t – 21, t – 28, and t – 35.

Relying on the analytical approach above, we observe that none of the strictly implemented NPIs under investigation had a substantial and consistent effect on COVID-19-attributed deaths over time (see Figure 1). None of the post-treatment trajectories differs significantly from the null-line. Even when comparing the post-treatment trajectories to the linear extrapolation of the 35-days pre-treatment period (dashed line), only strict stay-at-home requirements produce borderline-significant differences. We only observe a tentative change in the trend of COVID-19-related deaths starting around 30 days after strict stay-at-home rules have been introduced, but this does not exert a statistically significant effect. Similarly, we observe a tentative change in the trend of COVID-19-related fatalities 30 days after workplaces have been closed. However, the effects are not statistically different from zero. If we take this as evidence for a mitigating effect depends on whether we only consider reductions in the total of COVID-19-related deaths as mitigation or whether we also consider the option of preventing exponential growth in COVID-19-related deaths—an assumption we cannot test adequately based on the available data (see also Discussion).

**Figure 2:**
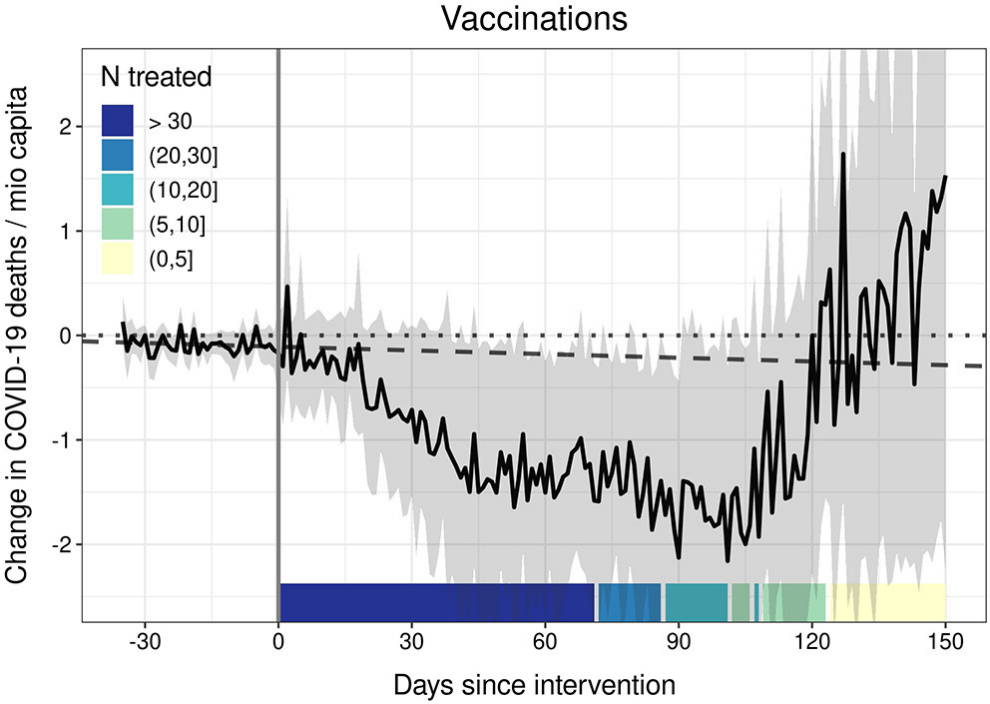
The effect of vaccinations (vaccine doses per inhabitant ≥80 %) on COVID-19 deaths. Generalized synthetic control estimator based on daily data. The black solid line represents the average treatment effect on the treated (ATT). Ribbons represent 95% non-parametric confidence intervals based on 1,000 bootstrap runs. The dotted line is the null line. The dashed line represents the linear prediction based on the 35 days before the intervention. Controls: 10 NPIs as stringency index, temperature, temperature^2^, cloud cover, precipitation, humidity, 7-day backwards rolling average of NPI-residualized COVID-19 cases at t – 7, t – 14, t – 21, t – 28, and t – 35.

We conducted a number of robustness checks (see Supplementary Material C). First, we carried out a proof-of-concept analysis to demonstrate that our model is able to detect a substantial and statistically significant ATT based on the data at hand: For this purpose, we investigated the effect of a pharmaceutical intervention (PI) instead of NPIs on fatalities. More specifically, we analyzed the impact of vaccinations against COVID-19 (27) on COVID-19-related deaths per 1 miocapita. Vaccination intervention is coded as treatment if the share of disseminated vaccine doses per capita exceeds 80%. Additionally, the model controls for all 10 NPIs under investigation (stringency index) and the controls listed above. As Figure 2 demonstrates, our model identifies a consistent and statistically significant mitigating effect of vaccination on COVID-19 fatalities from around day 45 to day 110 after treatment. This effect is also of substantial magnitude: for a country of 60 mio inhabitants, we estimate that vaccinations prevented around 90 death per day from 45 days onwards after reaching the threshold of 80 vaccinations per 100 inhabitants.

Second, we were concerned that spatial spillovers could affect our main results (42). Thus, we additionally controlled for NPI implementations in neighboring countries. The results indicate that spatial spillovers do not alter the results substantially on the national level (see Supplementary Figure 17). Third, we coded the highest two categories as intervention. Results (Supplementary Figure 18) do not indicate meaningful influences on fatalities, with the one exception of school closings. Note however that this result is rather unstable, as we lack a sufficient number of control units. Fourth, the results remain stable when controlling for the number of other interventions implemented instead of a stringency index (see Supplementary Figure 19). Fifth, we tested if results differ when using first-wave data only. Results with data before September 2020 (Supplementary Figure 20) deviate from our main findings only with regard to restrictions on internal movement. With first wave data, we observe a downturn in fatalities after restrictions on internal movement. However, the ATT is estimated with large insecurity. Sixth, we checked if our results depend on treatment timing. It is reasonable to assume that the implementation of NPIs in an early phase of the viral outbreak is more effective (19, 43). We therefore re-estimated our models separately for early and late adopters. We calculated the 7-day backwards rolling average of fatalities at treatment intervention and defined units below (above) the overall median as early (late) adopters. For early adopters (Supplementary Figure 21) the stay-at-home effect disappears, while it is stronger for late adopters (Supplementary Figure 22). This gives rise to the notion that stay-at-home rules have been implemented as a last resort to respond to a steep increase in cases. For all other NPIs the results are substantially similar.

## Discussion

Based on the applied GSC approach, we do not find substantial COVID-19-related fatality-reducing effects of the ten NPIs under investigation. We see a tentative change in the trend of COVID-19-related deaths starting around 30 days after stay-at-home rules have been introduced, and a slighter turn after workplace closing.

Our results do not corroborate the findings of former studies with less countries relying on first wave data, and following different, mostly DiD type analytical approaches on school closures (10, 13, 17–19), workplace closures (17–19), stay at home orders (16, 18), restrictions on international travel (15), and mask obligations (11, 15). These differences may also emerge from underreporting and missing timeliness of reporting of fatalities during the first viral outbreak (14, 17). Yet, our results mirror the findings of a recent study (32) following a conceptually similar analytical strategy to attenuate estimation bias stemming from self-selection into treatment while investigating the effect of 46 US state-level shelter-in-place orders on COVID-19 cases. In contrast to a DiD type approach, the study (32) finds no significant impacts of shelter-in-place orders on COVID-19 cases.

## Limitations

Nonetheless, our estimates might be subject to several issues. First, differences in population-wide compliance to NPIs might serve as an explanation for our findings (44, 45). This is more likely for mask obligations, contact tracing, testing policies, protecting elderly, and domestic travel restrictions. For instance, the tentative downwards trend after internal movement restrictions during the first wave (Supplementary Figure 20) may indicate higher compliance during this period compared to later periods. However, compliance rates are unlikely to serve as substantial explanation for the absence of mitigating effects of school and workplace closings, the closure of public transport, and restrictions on international travel. Second, our statistical approach complicates the investigation of simultaneous NPI implementations (46, 47). Comparing selected combinations of two simultaneous NPIs to countries without any of the two, however we do not find any consistent significant effect (Supplementary Figure 23).

Third, while the applied GSC approach attenuates the potential bias of self-selection into NPI implementation (pre-treatment), it cannot rule out the possibility of a potential post-treatment exponential growth in fatalities on our estimates. In other words, we might assume that these countries implementing the measures would otherwise have had an exponential growth in fatalities, and implementing these measures kept them relatively close to pre-treatment levels. Based on the available data, however, we cannot test this assumption.

Hence, our finding of statistically insignificant effects does not necessarily contradict the notion that the implementation of NPIs might have contributed to mitigate COVID-19-related fatalities by preventing exponential growth (rather than a decline in total COVID-19-related deaths).

Furthermore, data quality and local context might influence our results: There might still be misreporting of COVID-19-related deaths in non-hospitalized fatalities (20). The local context associated with the specific implementation of NPIs (e.g., school closure) might influence our results, as the relatively broad categories of OxCGRT might not capture variation in implementation, e.g., across subnational territories (21).

Nevertheless, we demonstrate that high vaccination rates against COVID-19 help to reduce fatalities while controlling for the implementation of 10 NPIs. This highlights the importance of PIs to combat the ongoing COVID-19 pandemic. However, the effectivity of vaccines hinges on continuous collaborative efforts to adapt COVID-19 vaccines given the spread of new variants (48) and on an increase in vaccination rates (49). The differential effectivity of NPIs and PIs might be related to the pronounced age gradient in COVID-19 related fatalities and associated multi-morbidities (50). This might especially explain why we find effects of workplace closing, public transport closing and contact tracing on COVID-19 cases (Supplementary Figure 24), but do not see significant effects on COVID-19 fatalities.

Altogether, the present study enhances the literature on the effectiveness of NPIs with respect to the period, the number of countries, and the analytical approach. However, some limitations associated with data quality and availability as well as the analytical strategy remain to be addressed by future research. Hence, we cannot test if the NPIs might have prevented a potential exponential growth in COVID-19 fatalities in those countries who selected into treatment. Still, the results of our study add new insights on the public health effects of NPIs and can thus help to guide future responses to pandemic outbreaks.

## Data Availability Statement

Publicly available datasets were analyzed in this study. This data can be found here: https://ourworldindata.org/coronavirus; https://www.bsg.ox.ac.uk/research/research-projects/covid-19-government-response-tracker.

## Author Contributions

SM and TR contributed equally to conceptualization, methodology, data analysis, and writing of the manuscript. Both authors have read and agreed to the final version of the manuscript.

## Funding

Open access funding was provided by the University Of Bern.

## Code Availability

All R code used to produce the reported results are available on Github: https://github.com/ruettenauer/Replication-npi-covid.

## Conflict of Interest

The authors declare that the research was conducted in the absence of any commercial or financial relationships that could be construed as a potential conflict of interest.

## Publisher's Note

All claims expressed in this article are solely those of the authors and do not necessarily represent those of their affiliated organizations, or those of the publisher, the editors and the reviewers. Any product that may be evaluated in this article, or claim that may be made by its manufacturer, is not guaranteed or endorsed by the publisher.
