# Erratum: The effects of non-pharmaceutical interventions on COVID-19 mortality: A generalized synthetic control approach across 169 countries

**DOI:** 10.3389/fpubh.2023.1186935

**Published:** 2023-03-27

**Authors:** Frontiers Production Office

**Affiliations:** Frontiers Media SA, Lausanne, Switzerland

**Keywords:** COVID-19, global public health, health policy, non-pharmaceutical interventions, lockdown, vaccination

An omission to the funding section of the original article was made in error. The following sentence has been added: “Open access funding was provided by the University Of Bern”.

The original article has been updated.

