# Supplementary material for: The Effects of Non-pharmaceutical Interventions on COVID-19 Mortality: A Generalized Synthetic Control Approach Across 169 Countries
Source: Front Public Health. 2022 Apr 4;10:820642. doi: 10.3389/fpubh.2022.820642 (PMC9013850; doi:10.3389/fpubh.2022.820642)
Supplement: Supplementary file 1 [file Data_Sheet_1.pdf]

Supplementary material

A Descriptives

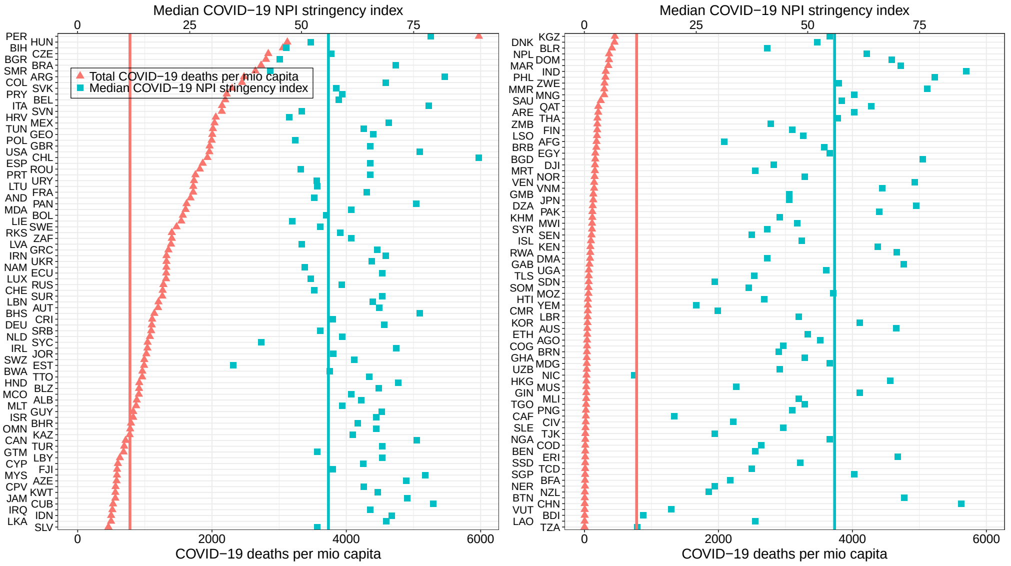

Figure S1: Total COVID-19 deaths per million capita and median NPI stringency index per country. Countries are descending order of total COVID-19 deaths per capita.

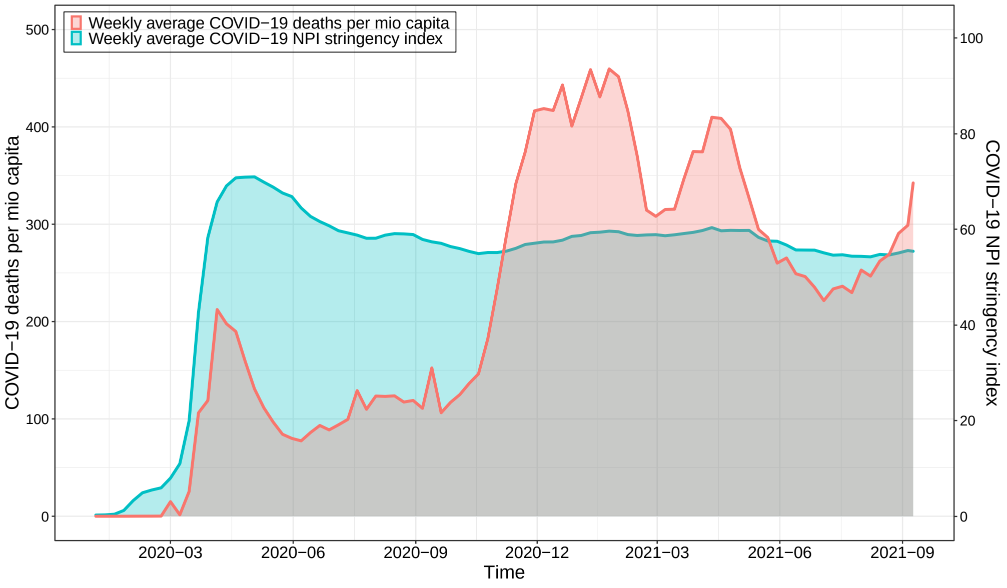

Figure S2: Average COVID-19 deaths per week and average NPI stringency index of all countries over time.

B Treatment Sequences

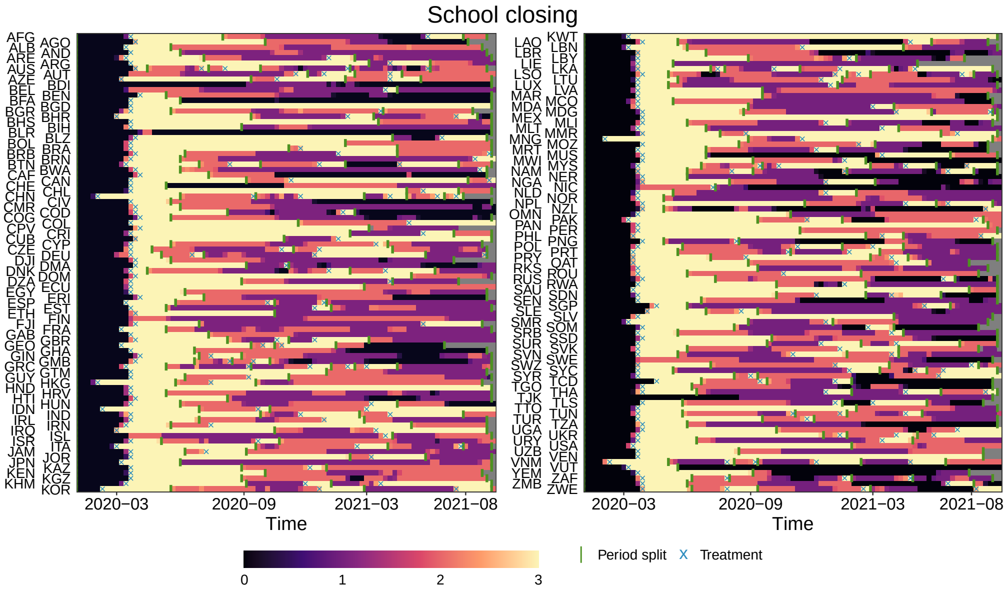

Figure S3: School closing treatment sequence, based on weekly average data. Green dashes indicate id-period splits. Blue crosses indicate the week of initial treatment. Note: 0 - no measures; 1 - recommend closing or all schools open with alterations resulting in significant differences compared to non-COVID-19 operations; 2 - require closing (only some levels or categories, eg just high school, or just public schools); 3 - require closing all levels; grey - no data.

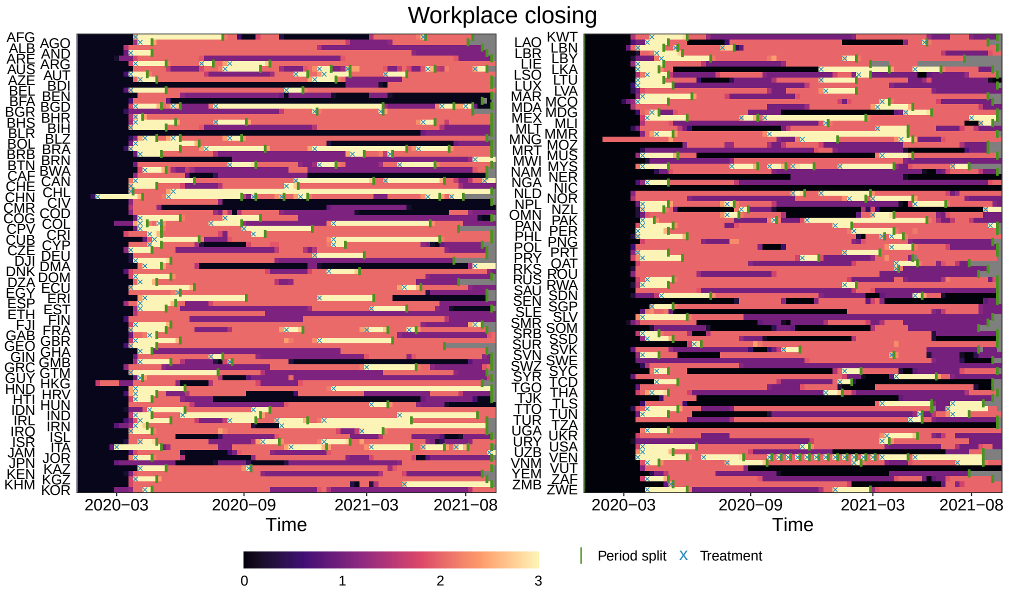

Figure S4: Workplace closing treatment sequence, based on weekly average data. Green dashes indicate id-period splits. Blue crosses indicate the week of initial treatment. Note: 0 - no measures; 1 - recommend closing (or recommend work from home); 2 - require closing (or work from home) for some sectors or categories of workers; 3 - require closing (or work from home) for all-but-essential workplaces (eg grocery stores, doctors); grey - no data.

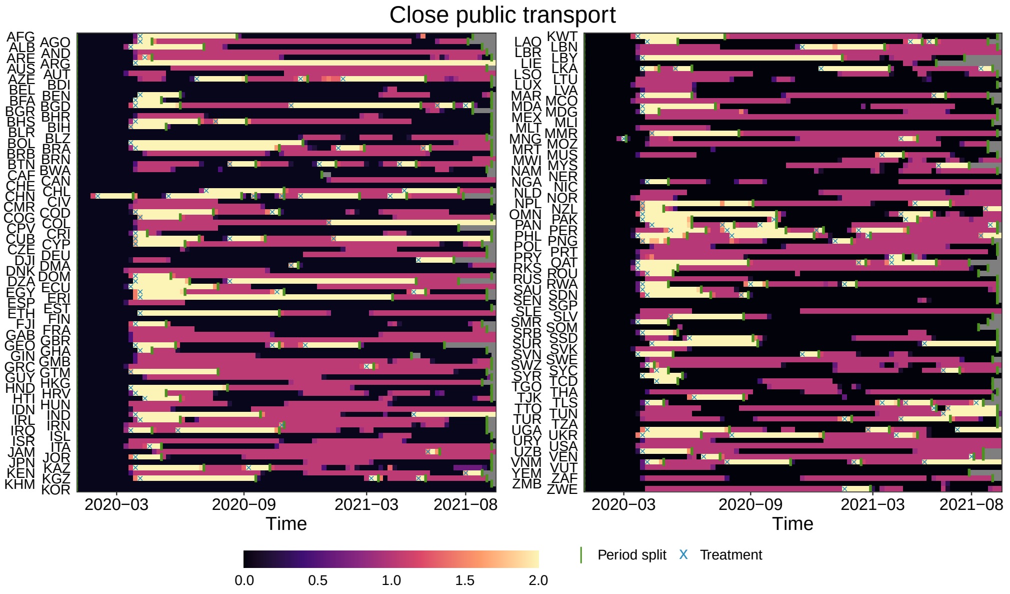

Figure S5: Close public transport treatment sequence, based on weekly average data. Green dashes indicate id-period splits. Blue crosses indicate the week of initial treatment. Note: 0 - no measures; 1 - recommend closing (or significantly reduce volume/route/means of transport available); 2 - require closing (or prohibit most citizens from using it); grey - no data.

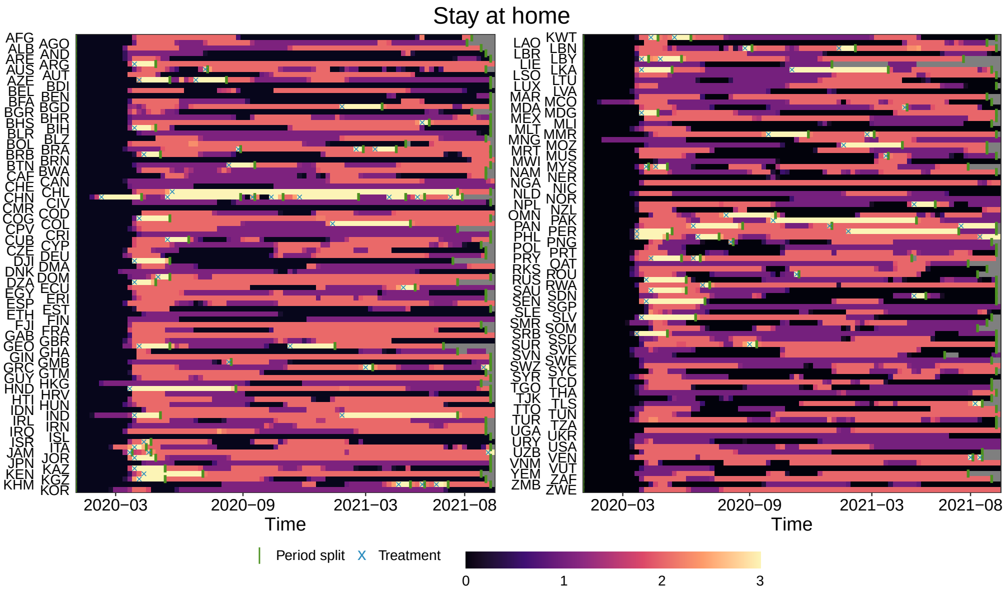

Figure S6: Stay at home treatment sequence, based on weekly average data. Green dashes indicate id-period splits. Blue crosses indicate the week of initial treatment. Note: 0 - no measures; 1 - recommend not leaving house; 2 - require not leaving house with exceptions for daily exercise, grocery shopping, and 'essential' trips; 3 - require not leaving house with minimal exceptions (eg allowed to leave once a week, or only one person can leave at a time, etc); grey - no data.

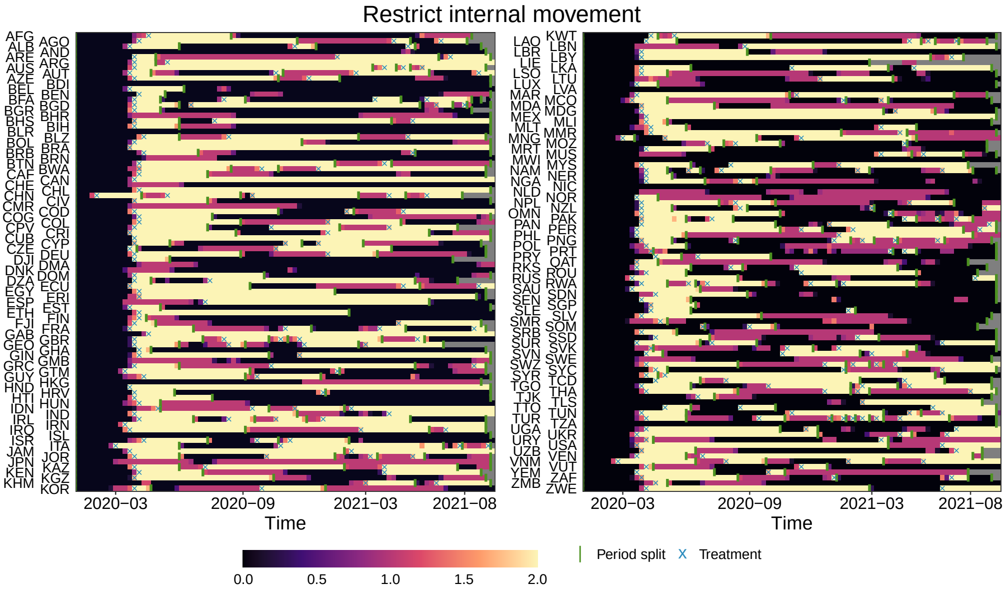

Figure S7: Restrict internal movement treatment sequence, based on weekly average data. Green dashes indicate id-period splits. Blue crosses indicate the week of initial treatment. Note: 0 - no measures; 1 - recommend not to travel between regions/cities; 2 - internal movement restrictions in place; grey - no data.

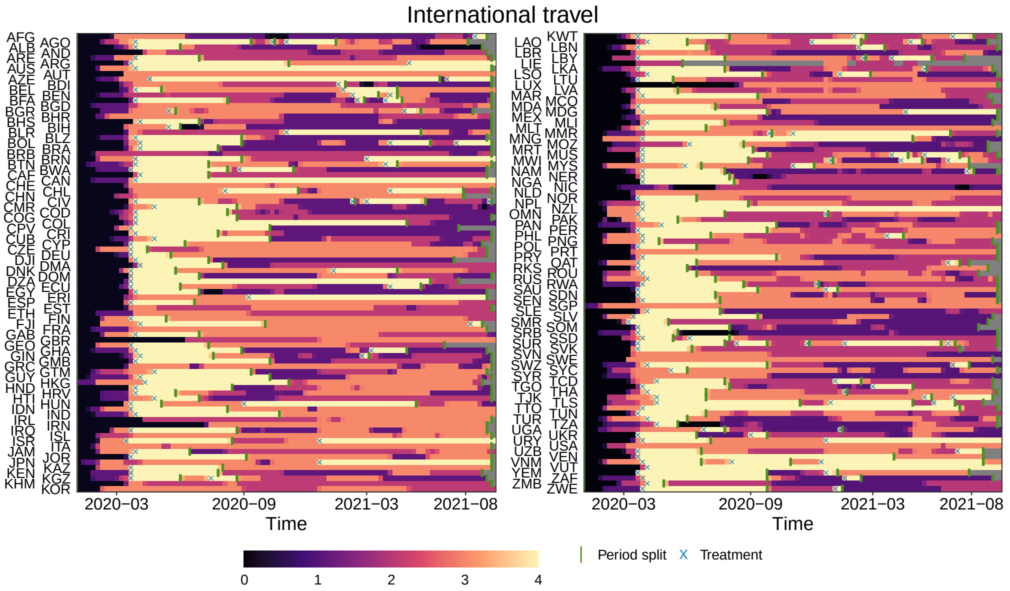

Figure S8: International travel treatment sequence, based on weekly average data. Green dashes indicate id-period splits. Blue crosses indicate the week of initial treatment. Note: 0 - no restrictions; 1 - screening arrivals; 2 - quarantine arrivals from some or all regions; 3 - ban arrivals from some regions; 4 - ban on all regions or total border closure; grey - no data.

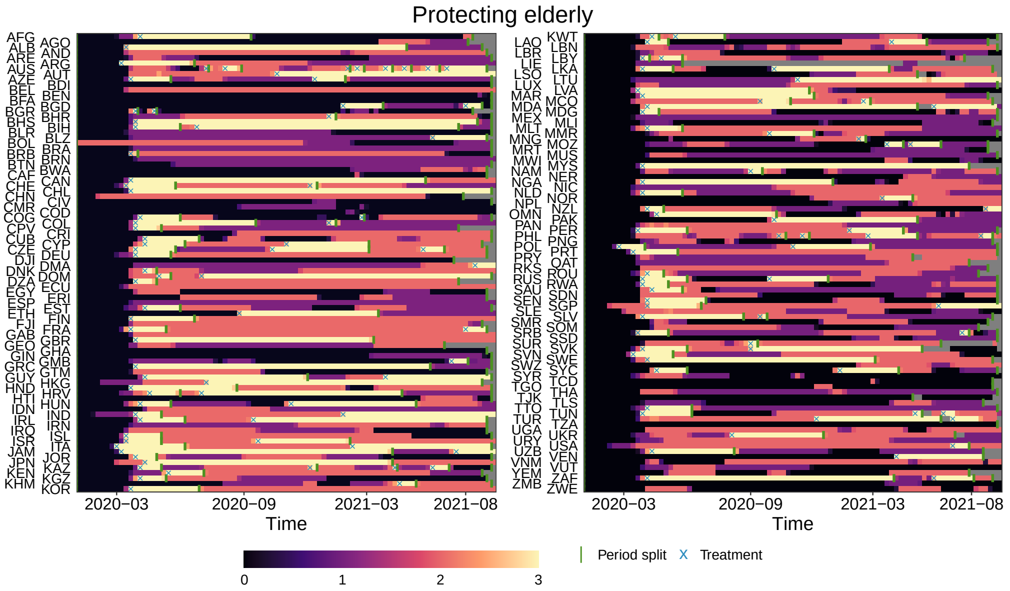

Figure S9: Protecting elderly treatment sequence, based on weekly average data. Green dashes indicate id-period splits. Blue crosses indicate the week of initial treatment. Note: 0 - no measures; 1 - Recommended isolation, hygiene, and visitor restriction measures in LTCFs and/or elderly people to stay at home; 2 - Narrow restrictions for isolation, hygiene in long-term care facilities (LTCFs), some limitations on external visitors and/or restrictions protecting elderly people at home; 3 - Extensive restrictions for isolation and hygiene in LTCFs, all non-essential external visitors prohibited, and/or all elderly people required to stay at home and not leave the home with minimal exceptions, and receive no external visitors; grey - no data.

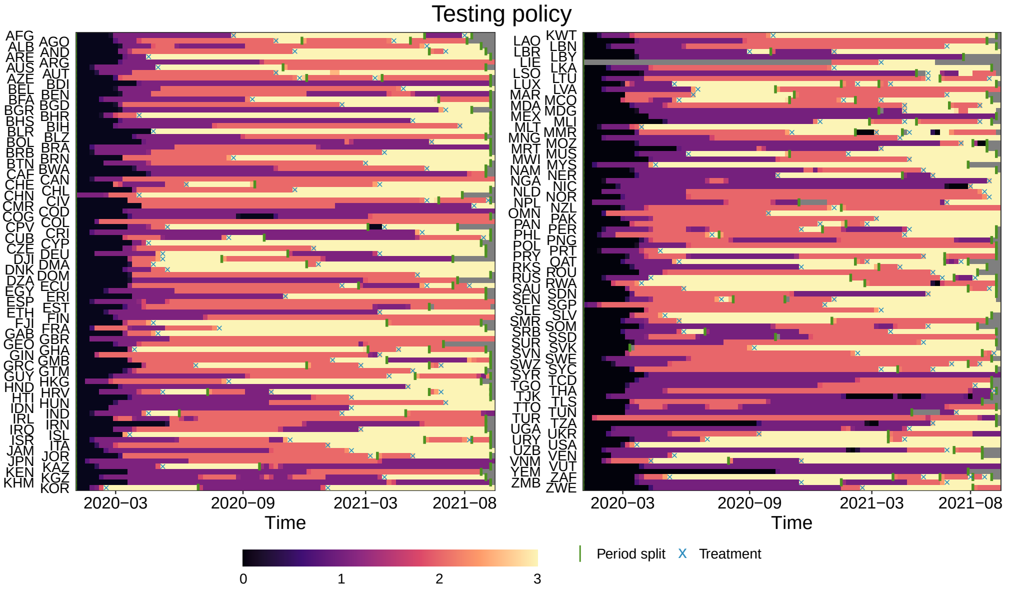

Figure S10: Testing policy treatment sequence, based on weekly average data. Green dashes indicate id-period splits. Blue crosses indicate the week of initial treatment. Note: 0 - no testing policy; 1 - only those who both (a) have symptoms AND (b) meet specific criteria (eg key workers, admitted to hospital, came into contact with a known case, returned from overseas); 2 - testing of anyone showing COVID-19 symptoms; 3 - open public testing (eg ”drive through” testing available to asymptomatic people); grey - no data.

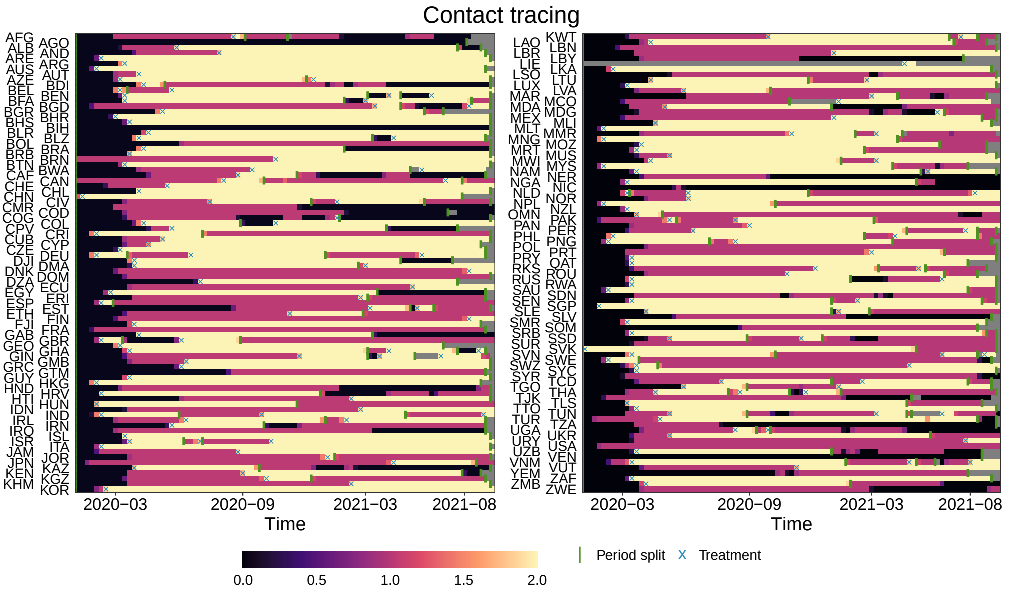

Figure S11: Contact tracing treatment sequence, based on weekly average data. Green dashes indicate id-period splits. Blue crosses indicate the week of initial treatment. Note: 0 - no contact tracing; 1 - limited contact tracing; not done for all cases; 2 - comprehensive contact tracing; done for all identified cases; grey - no data.

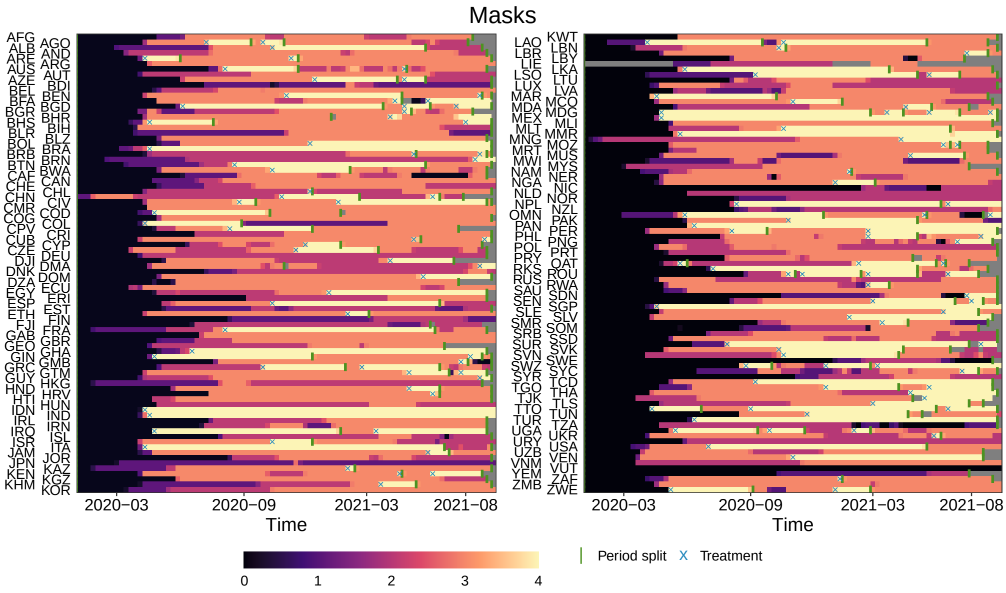

Figure S12: Masks treatment sequence, based on weekly average data. Green dashes indicate id-period splits. Blue crosses indicate the week of initial treatment. Note: 0 - No policy; 1 - Recommended; 2 - Required in some specified shared/public spaces outside the home with other people present, or some situations when social distancing not possible; 3 - Required in all shared/public spaces outside the home with other people present or all situations when social distancing not possible; 4 - Required outside the home at all times regardless of location or presence of other people; grey - no data.

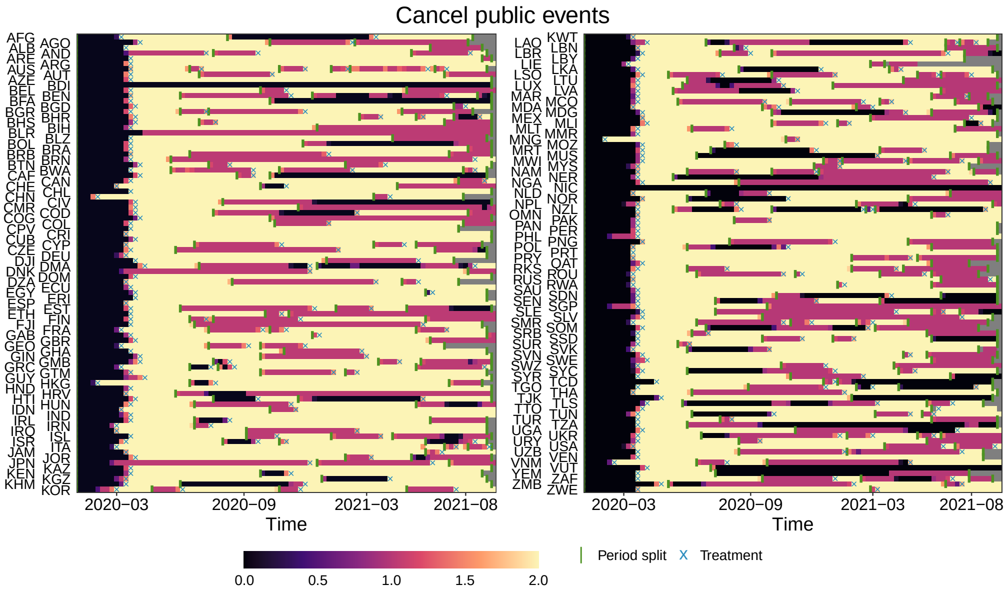

Figure S13: Cancel public events treatment sequence, based on weekly average data. Green dashes indicate id-period splits. Blue crosses indicate the week of initial treatment. Note: 0 - no measures; 1 - recommend cancelling; 2 - require cancelling; grey - no data.

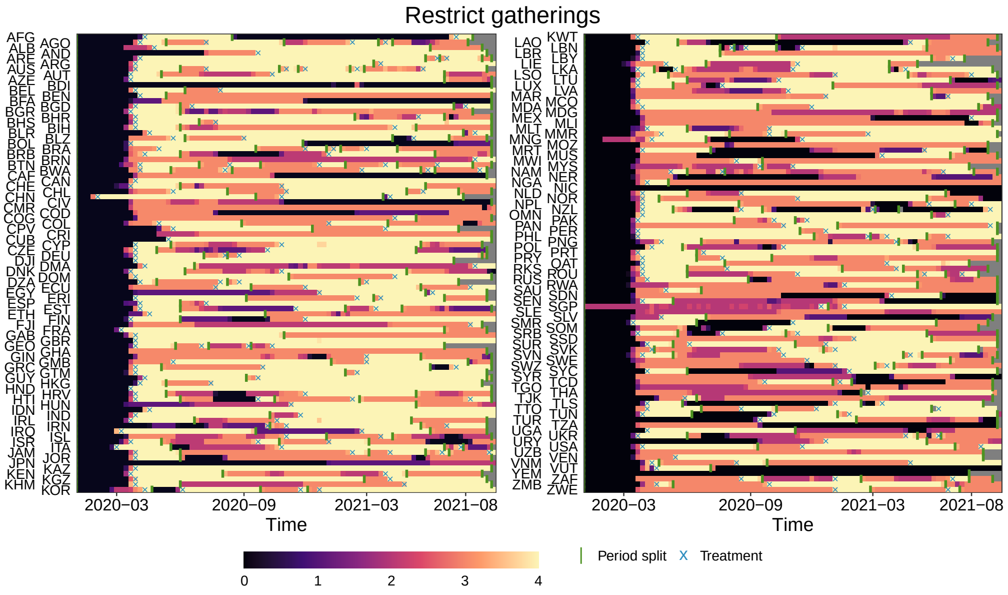

Figure S14: Restrict gatherings treatment sequence, based on weekly average data. Green dashes indicate id-period splits. Blue crosses indicate the week of initial treatment. Note: 0 - no restrictions; 1 - restrictions on very large gatherings (the limit is above 1000 people); 2 - restrictions on gatherings between 101-1000 people; 3 - restrictions on gatherings between 11-100 people; 4 - restrictions on gatherings of 10 people or less; grey - no data.

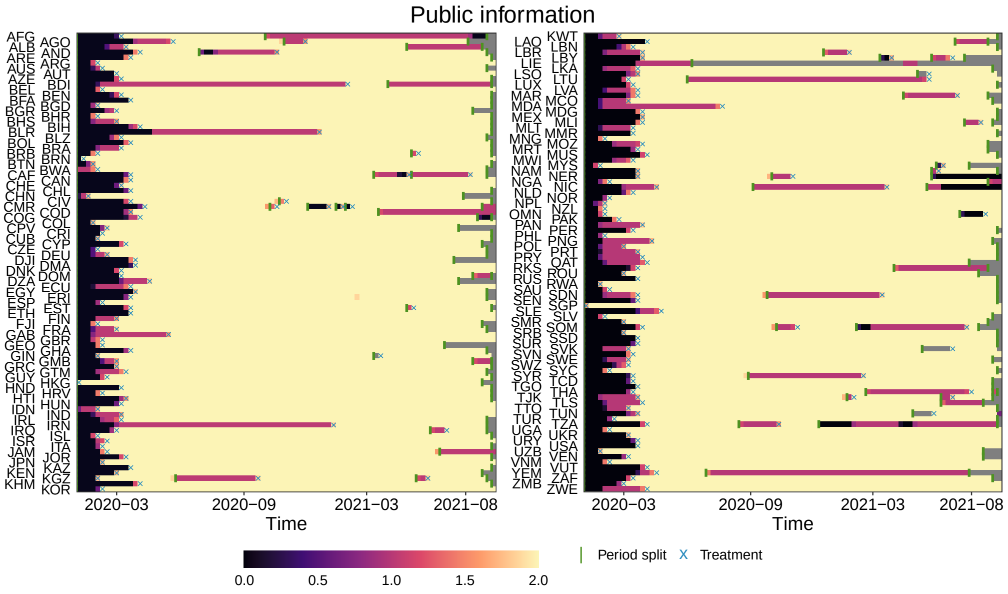

Figure S15: Public information campaigns treatment sequence, based on weekly average data. Green dashes indicate id-period splits. Blue crosses indicate the week of initial treatment. Note: 0 - no Covid-19 public information campaign; 1 - public officials urging caution about Covid-19; 2- coordinated public information campaign (eg across traditional and social media); grey - no data.

C Robustness checks

C.1 Without controlling for cases

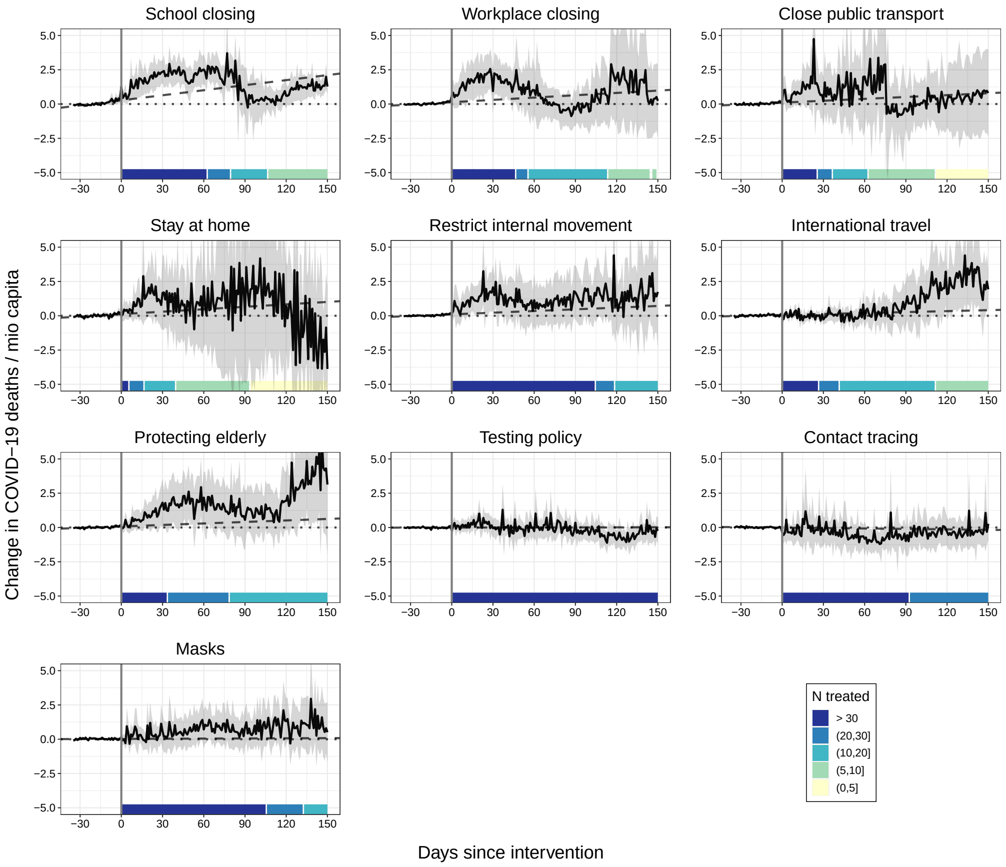

Figure S16: The effect of NPIs on COVID-19 deaths, omitting the residualized COVID-19 cases as control. Generalized synthetic control estimator based on daily data. Black solid lines represent the average treatment effects on the treated (ATTs). Ribbons represent 95% non-parametric confidence intervals based on 1,000 bootstrap runs, clustered at the country level. Dotted lines are the null lines. Dashed lines represent linear predictions based on the 35 days before the intervention. Controls: 9 remaining NPIs as stringency index, temperature, temperature^2, cloud cover, precipitation, humidity, total vaccinations.

C.2 Spatial spillovers

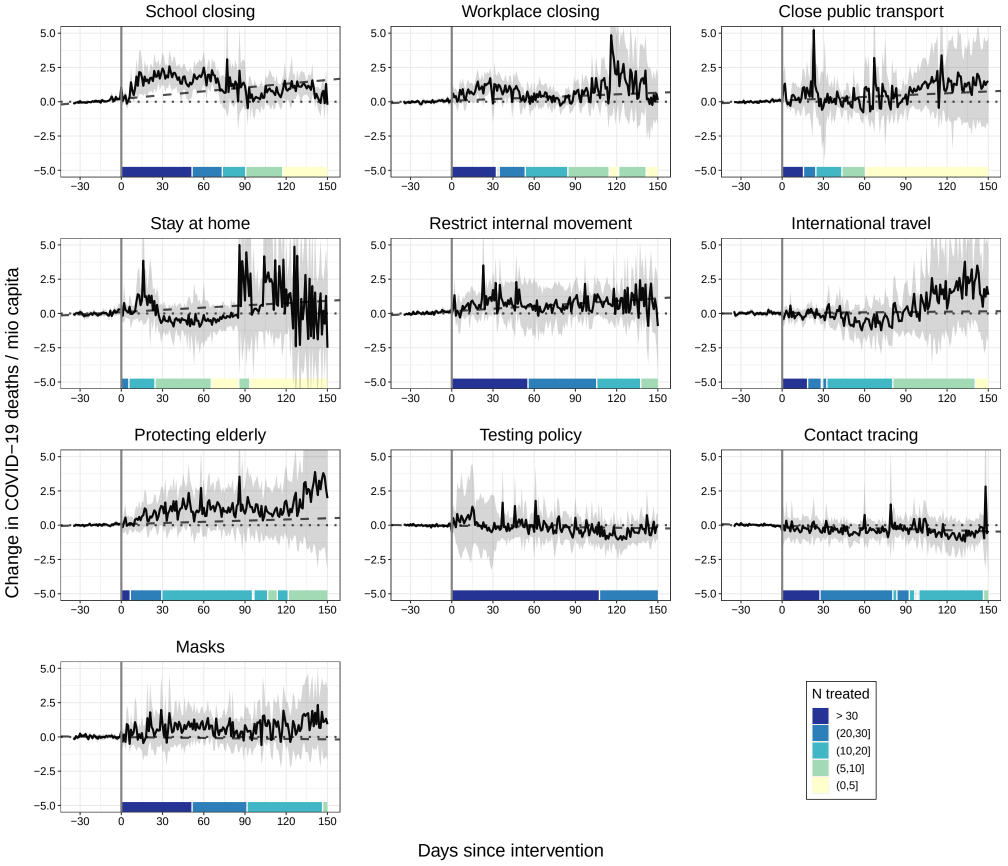

Figure S17: The effect of NPIs on COVID-19 deaths, additionally controlling for interventions in neighbouring countries. Generalized synthetic control estimator based on daily data. Black solid lines represent the average treatment effects on the treated (ATTs). Ribbons represent 95% non-parametric confidence intervals based on 1,000 bootstrap runs, clustered at the country level. Dotted lines are the null lines. Dashed lines represent linear predictions based on the 35 days before the intervention. Controls: 9 remaining NPIs as stringency index, average of all 10 NPIs as stringency index in adjacent countries, temperature, temperature^2, cloud cover, precipitation, humidity, total vaccinations, 7-day backwards rolling average of NPI-residualized COVID-19 cases at $t-7$, $t-14$, $t-21$, $t-28$, and $t-35$.

C.3 Highest two NPI categories

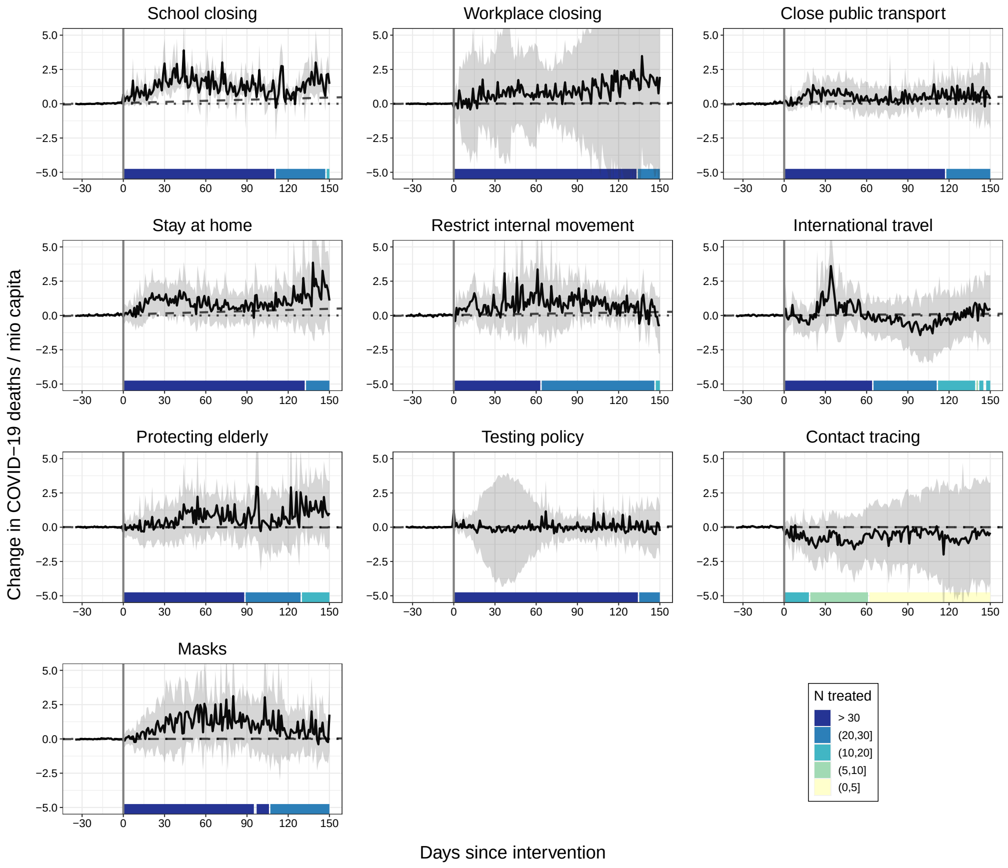

Figure S18: The effect of NPIs on COVID-19 deaths, coding the highest two categories as intervention. Generalized synthetic control estimator based on daily data. Black solid lines represent the average treatment effects on the treated (ATTs). Ribbons represent 95% non-parametric confidence intervals based on 1,000 bootstrap runs, clustered at the country level (note: for contact tracing, SEs are not clustered because of the low number of clusters). Dotted lines are the null lines. Dashed lines represent linear predictions based on the 35 days before the intervention. Controls: 9 remaining NPIs as stringency index, temperature, temperature^2, cloud cover, precipitation, humidity, total vaccinations, 7-day backwards rolling average of NPI-residualized COVID-19 cases at $t-7$, $t-14$, $t-21$, $t-28$, and $t-35$.

C.4 Number of other interventions

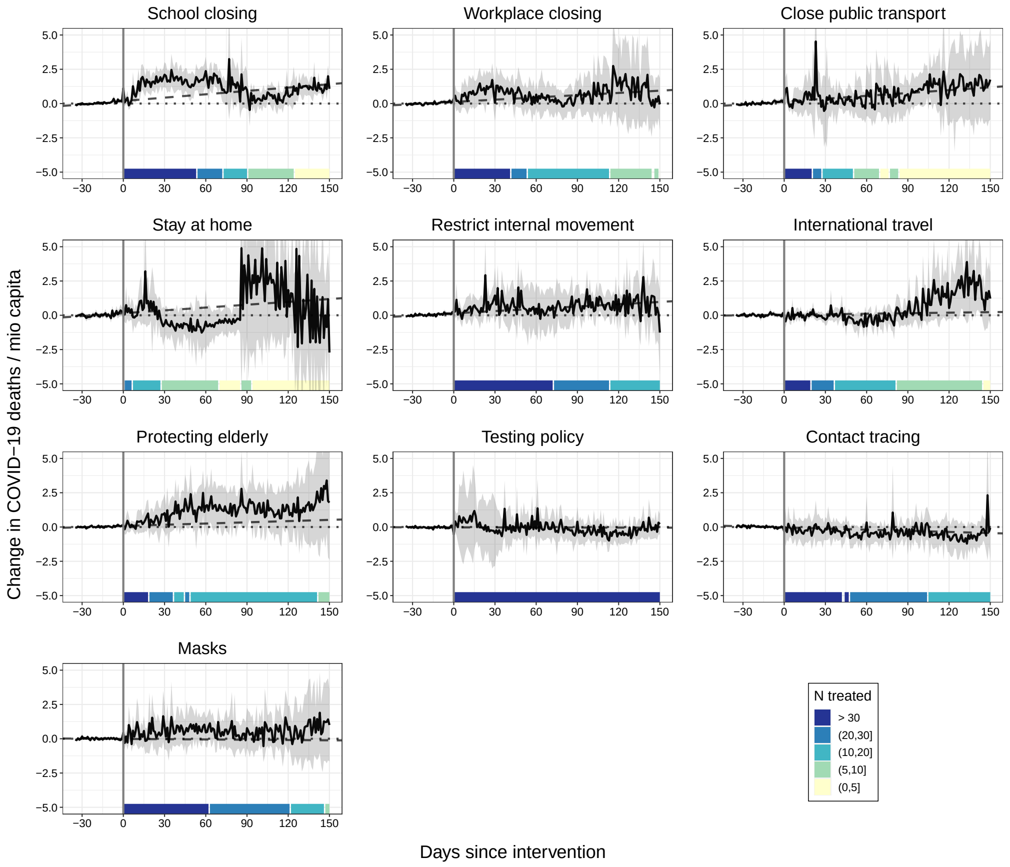

Figure S19: The effect of NPIs on COVID-19 deaths, controlling for the number of other interventions instead of stringency index. Generalized synthetic control estimator based on daily data. Black solid lines represent the average treatment effects on the treated (ATTs). Ribbons represent 95% non-parametric confidence intervals based on 1,000 bootstrap runs, clustered at the country level. Dotted lines are the null lines. Dashed lines represent linear predictions based on the 35 days before the intervention. Controls: number of other NPIs (dichotomized), temperature, temperature^2, cloud cover, precipitation, humidity, total vaccinations, 7-day backwards rolling average of NPI-residualized COVID-19 cases at $t-7$, $t-14$, $t-21$, $t-28$, and $t-35$.

C.5 First wave data only (until August 31, 2020)

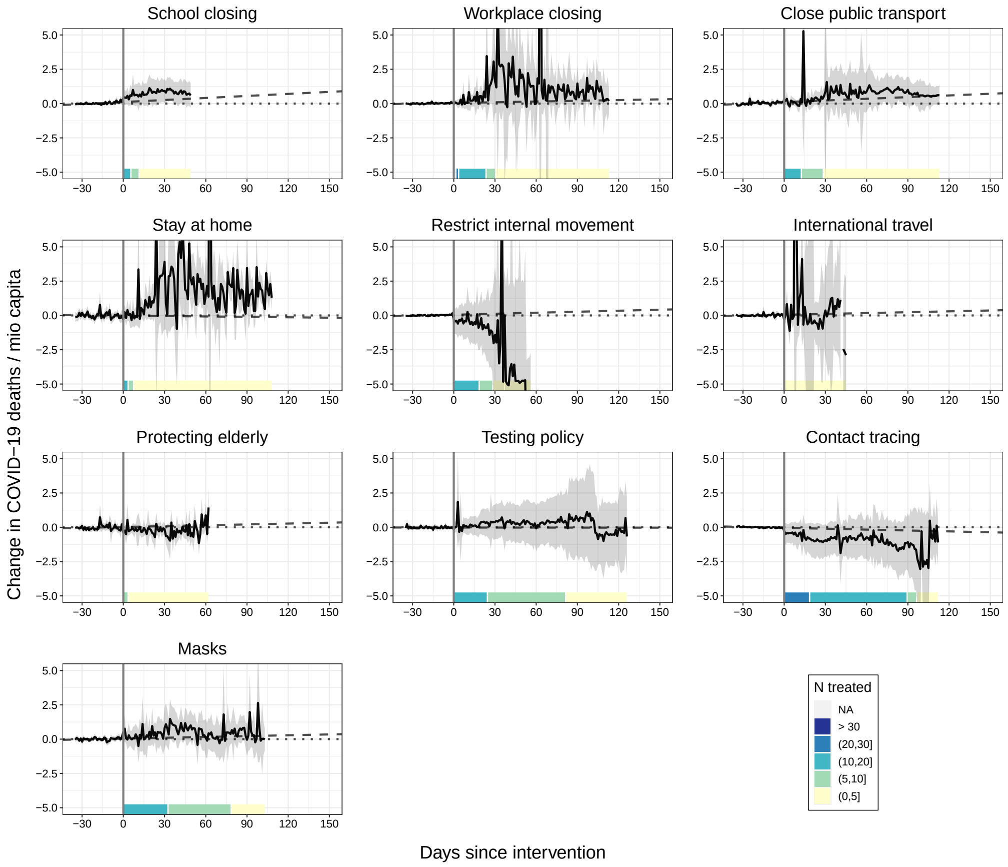

Figure S20: The effect of NPIs on COVID-19 deaths based on data until August 31, 2020. Generalized synthetic control estimator based on daily data. Black solid lines represent the average treatment effects on the treated (ATTs). Ribbons represent 95% non-parametric confidence intervals based on 1,000 bootstrap runs (not clustered because of low N clusters). Dotted lines are the null lines. Dashed lines represent linear predictions based on the 35 days before the intervention. Controls: 9 remaining NPIs as stringency index, temperature, temperature^2, cloud cover, precipitation, humidity, total vaccinations, 7-day backwards rolling average of NPI-residualized COVID-19 cases at $t-7$, $t-14$, $t-21$, $t-28$, and $t-35$.

C.6 Early and late adopters

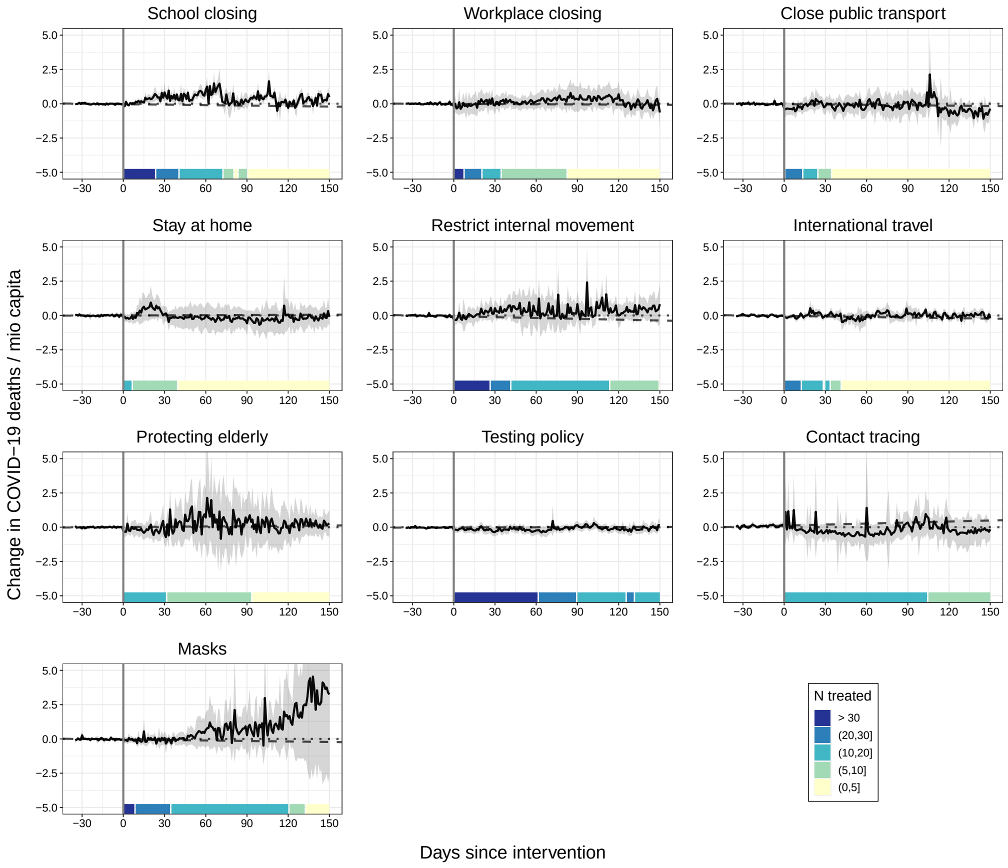

Figure S21: Early adopters (number of deaths at implementation below median): The effect of NPIs on COVID-19 deaths. Generalized synthetic control estimator based on daily data. Black solid lines represent the average treatment effects on the treated (ATTs). Ribbons represent 95% non-parametric confidence intervals based on 1,000 bootstrap runs, clustered at the country level. Dotted lines are the null lines. Dashed lines represent linear predictions based on the 35 days before the intervention. Controls: 9 remaining NPIs as stringency index, temperature, temperature^2, cloud cover, precipitation, humidity, total vaccinations, 7-day backwards rolling average of NPI-residualized COVID-19 cases at $t-7$, $t-14$, $t-21$, $t-28$, and $t-35$.

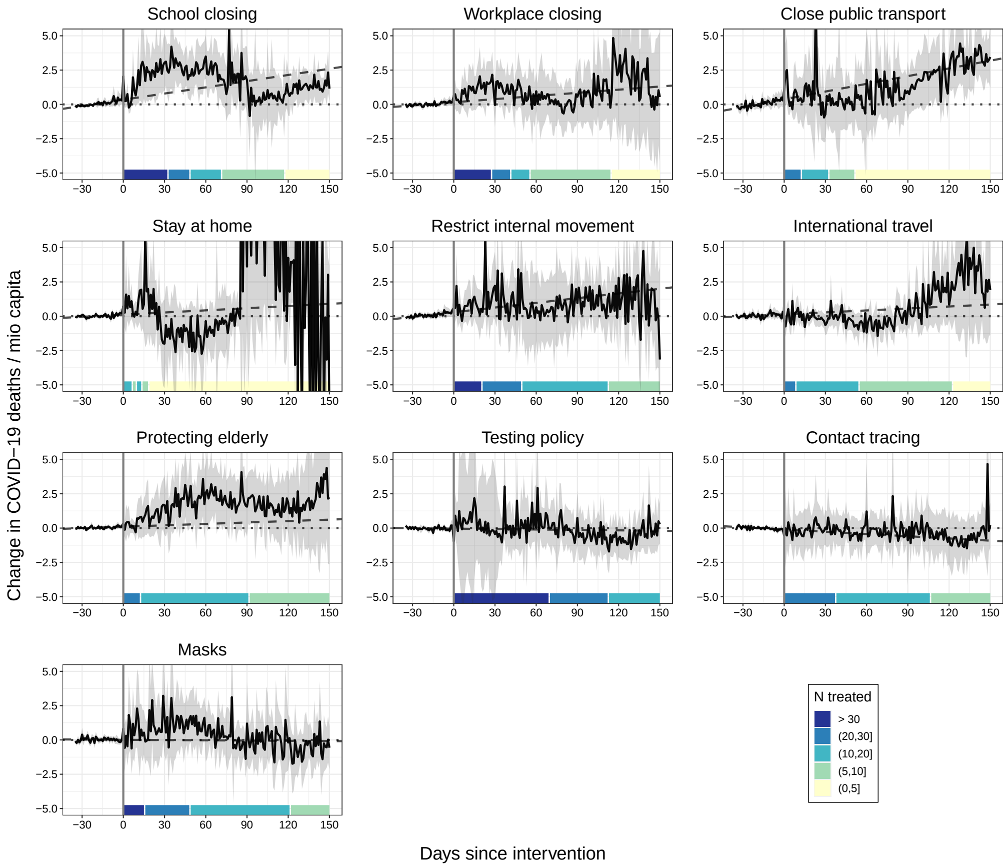

Figure S22: Late adopters (number of deaths at implementation above median): The effect of NPIs on COVID-19 deaths. Generalized synthetic control estimator based on daily data. Black solid lines represent the average treatment effects on the treated (ATTs). Ribbons represent 95% non-parametric confidence intervals based on 1,000 bootstrap runs, clustered at the country level. Dotted lines are the null lines. Dashed lines represent linear predictions based on the 35 days before the intervention. Controls: 9 remaining NPIs as stringency index, temperature, temperature^2, cloud cover, precipitation, humidity, total vaccinations,7-day backwards rolling average of residualized COVID-19 cases at $t-7$, $t-14$, $t-21$, $t-28$, and $t-35$.

C.7 NPI combinations

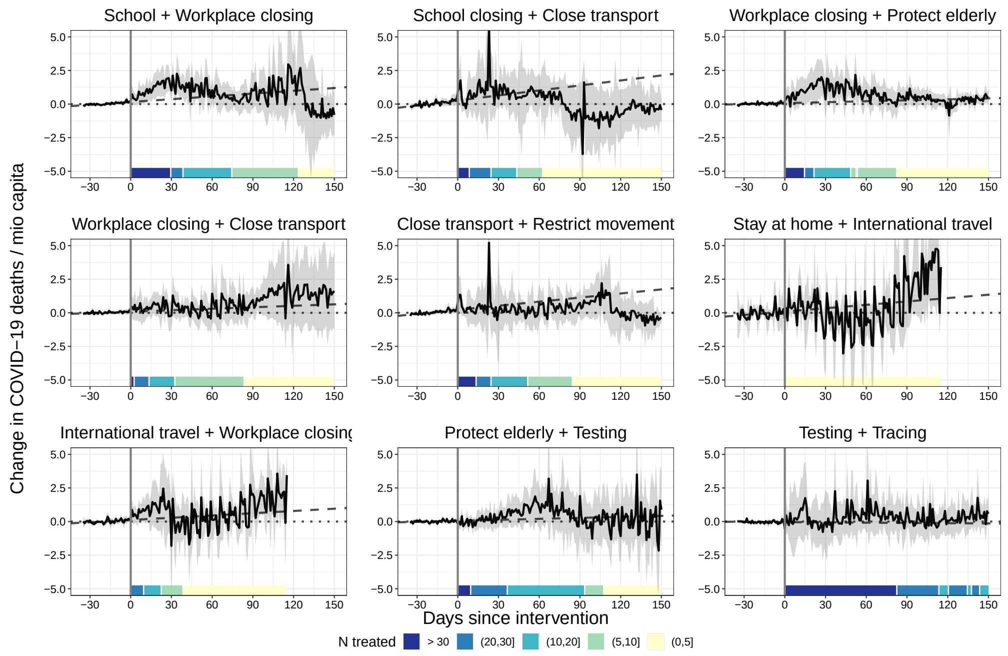

Figure S23: The effect of NPI combinations on COVID-19 deaths compared to countries having none of the respective NPIs implemented. Generalized synthetic control estimator based on daily data. Black solid lines represent the average treatment effects on the treated (ATTs). Ribbons represent 95% non-parametric confidence intervals based on 1,000 bootstrap runs, clustered at the country level. Dotted lines are the null lines. Dashed lines represent linear predictions based on the 35 days before the intervention. Controls: 9 remaining NPIs as stringency index, temperature, temperature^2, cloud cover, precipitation, humidity, total vaccinations, 7-day backwards rolling average of residualized COVID-19 cases at $t-7$, $t-14$, $t-21$, $t-28$, and $t-35$.

D Analysis of cases

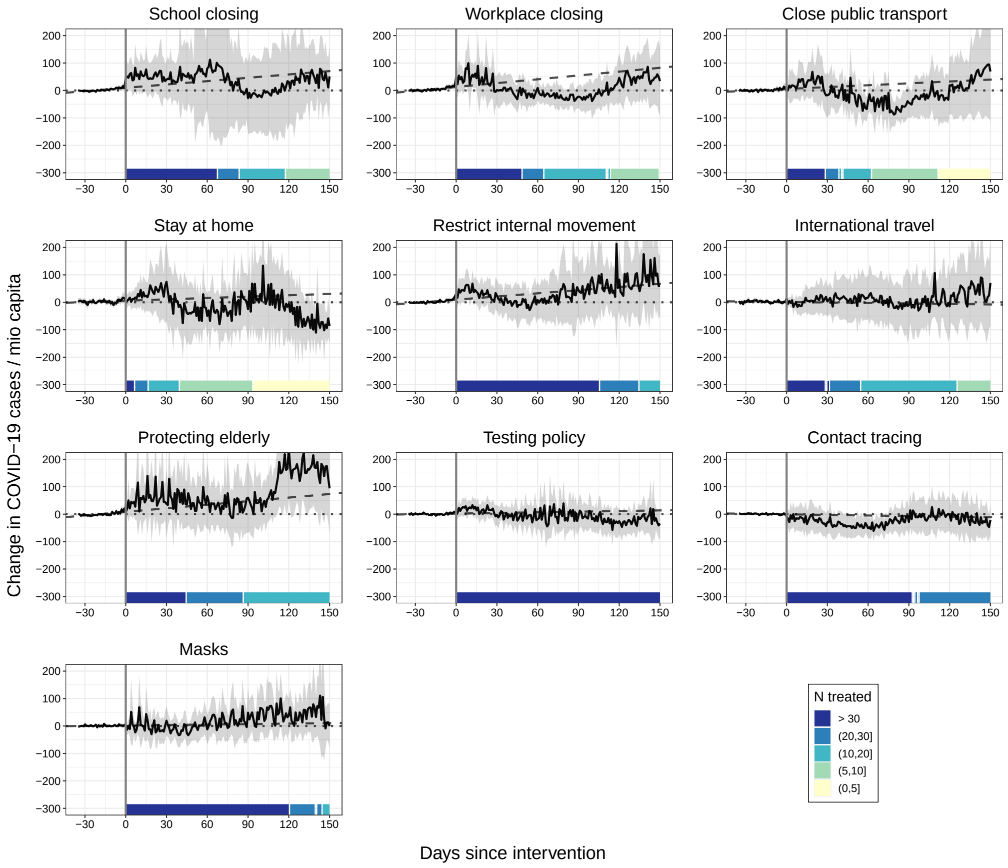

Figure S24: The effect of NPIs on COVID-19 cases. Generalized synthetic control estimator based on daily data. Black solid lines represent the average treatment effects on the treated (ATTs). Ribbons represent 95% non-parametric confidence intervals based on 1,000 bootstrap runs, clustered at the country level. Dotted lines are the null lines. Dashed lines represent linear predictions based on the 35 days before the intervention. Controls: 9 remaining NPIs as stringency index, temperature, temperature^2, cloud cover, precipitation, total vaccinations.

E Literature review

Table S1: Overview of empirical studies on the effectiveness of non-pharmaceutical interventions (NPIs) against COVID-19

Study	Outcome	Pop size	NPIs	Territories	Time span	Time dimension	Method	Time lags	Results
[1]	infections	yes	OxCGRT	175 countries	?	days	multiple-event model	yes (10 days pre-treatment, 45 days post-treatment)	Mitigating effect for cancel public events, restrictions on gatherings, school closure, workplace closure, and perhaps stay-at-home requirements
[2]	infections, deaths	yes	school closure	US	9 March ? 7 May 2020	days	Interrupted time series analysis	yes	Large mitigating effect.
[3]	infections	yes	many, self-coded	20 countries	-15 April 2020	days	?	yes	Mitigating effect for workplace closure, restrictions on gatherings, international travel, cancel public events, school closure.
[4].	infections	yes	OxCGRT	10 countries	February-April 2020	days	Fixed effects regression	no	Mitigating effect for all, but no difference between less restrictive and more restrictive (stay-at-home, business closure) NPIs

Table S1: Overview of empirical studies on the effectiveness of non-pharmaceutical interventions (NPIs) against COVID-19

Study	Outcome	Pop size	NPIs	Territories	Time span	Time dimension	Method	Time lags	Results
[5]	Rt	na	self-coded, different measures, limited to: limit gatherings, school closure, workplace closure, stay-at-home order.	41 countries	January-May 2020	days	Semi-mechanistic Bayesian hierarchical model (Flaxman et al. 2020)	yes	School closure, limit gatherings to max. 10 persons, and workplace closure reduce transmission. Other NPIs are not substantially effective.
[6]	infections	yes	shelter-in-place orders	46 US states	March – April 2020	days	unconfoundedness type SIRD model	yes	no mitigating effect
[7]	cases	no	OxCGRT (11 of them)	166 countries	January-May 2020	days	First difference regression	Yes	Mitigating effects for school closures, workplace closures, cancelation of events, restrictions on gatherings, stay at home orders, and restrictions on domestic movement. Increasing effects for public information, testing and tracing.
[8]	infections, recoveries, deaths	no	travel restriction, mask wearing, lock-down, social distancing, school closure, and centralized quarantine	9 countries	January-April 2020	days	dynamic panel SIR	yes	Mitigating effect of centralized quarantine, lockdown, school closure, and wearing masks.
[9]	cases, deaths	yes	Raifman et al. (2020)	US states	March-August 2020	days	Structural equation model	yes	Robust mitigating effect of masks

Table S1: Overview of empirical studies on the effectiveness of non-pharmaceutical interventions (NPIs) against COVID-19

Study	Outcome	Pop size	NPIs	Territories	Time span	Time dimension	Method	Time lags	Results
[10]	infections	na	Travel restrictions	China	January-February 2020	days	Global Epidemic and Mobility Model	yes	Mitigating effect of travel restrictions
[11]	infections	?	Social distancing, face masks, eye protection	172 bservational studies from 22 countries	-June 2020	na	Random effects meta regression	?	Mitigating effect of social distancing (>1m), face masks, and eye protection
[12]	infections, Rt	na	many	Hong Kong	January-March 2020	days	Changes	?	Mitigating effect
[13]	infections, deaths	yes	Lockdown	China (2 provinces)	Jan-20	days	Interrupted time series model	yes	Mitigating effect
[14]	infections	na	mild social distancing, strong social distancing, and contact ban	Germany	March-April 2020	days	Bayesian SIR	yes	Mitigating effect
[15]	deaths	yes	OxCGRT	32 countries	January-April 2020	days	Time-series analysis approach	?	Mitigating effect of school closures
[16],	infections, Rt, deaths	no	Self-coded NPIs	11 countries	February-May 2020	days	Semi-mechanistic Bayesian hierarchical model	yes	Strong mitigating effect of 'complete lockdown'
[17]	infections	na	NPIs imposed	Italy	February-March 2020	days	SEIR model	yes	Strong mitigating effect

Table S1: Overview of empirical studies on the effectiveness of non-pharmaceutical interventions (NPIs) against COVID-19

Study	Outcome	Pop size	NPIs	Territories	Time span	Time dimension	Method	Time lags	Results
[18]	Rt	na	Many (Desvars-Larrive et al. 2020)	79 territories	March-April 2020	days	Case-control analysis, LASSO time-series regression, random forests, transformers.	yes	Large mitigating effects of small gathering cancellation, closure of educational institutions, border restrictions, increase availability of personal protective equipment, individual movement restrictions, and national lockdown. Other NPIs are not effective.
[19]	cumulated infections	no	OxCGRT (11 of them)	108 countries	January-June 2020	na	OLS	yes	Mitigating effect of school closure, restrictions on internal movement, contact tracing
[20]	infections	no	Many, self-coded	6 countries	January-April 2020	days	SIR	yes	Mitigating effect of all measures
[21]	IRR	yes	OxCGRT	149 countries	January-May 2020	days	Interrupted time series	yes	Mitigating effect of physical distancing interventions, but not for closure of public transport
[22]	incidence	yes	Self-coded	Germany (401 NUTS-3 regions)	February-April 2020	days	Spatial difference-in-differences model (Durbin)	yes	Mitigating effect of contact restrictions and school closure; and for masks in a few regions.
[23],	infections	no	Mobility restrictions	China	January-February 2020	days	Generalized linear model	yes	Strong mitigating effect of mobility restriction

Table S1: Overview of empirical studies on the effectiveness of non-pharmaceutical interventions (NPIs) against COVID-19

Study	Outcome	Pop size	NPIs	Territories	Time span	Time dimension	Method	Time lags	Results
[24]	infections	na	Various	China	January-April 2020	days	SEIR model	yes	Mitigating effect of tracing and isolation, smaller mitigating effect for contact reductions, travel restrictions
[25]	cumulated deaths	yes	OxCGRT	196 countries	January-May 2020	na	OLS	yes	Mitigating effect of masks, travel restrictions
[26]	Rt	na	OxCGRT	131 countries	January-July 2020	days	OLS	yes	Mitigating effect of cancel public events, increasing effect of the lifting of school reopening, and lifting bans on public gatherings of more than ten people.
[27]	Rt	na	13 NPIs from OxCGRT	130	January-June 2020	days	Panel regression	yes	Mitigating effect of school closure, internal movement restrictions
[28]	infections	no	Masks	16 US states	March-May 2020	days	difference-in-differences model	yes	Mitigating effect
[29]	infections	no	Conglomerate of NPIs	China	21 January-10 March 2020	days	SIR model	yes	Mitigating effect
[30]	infections	yes	Masks	Germany	March-April 2020	days	Synthetic control	yes	large mitigating effect
[31],	infections	yes	‘Lockdown’	Spanish provinces	1 March-4 April 2020	days	First Difference Regression (with spatial weights)	yes	Mitigating effect, spatial propagation between provinces

Table S1: Overview of empirical studies on the effectiveness of non-pharmaceutical interventions (NPIs) against COVID-19

Study	Outcome	Pop size	NPIs	Territories	Time span	Time dimension	Method	Time lags	Results
[32]	infections	no	Restriction of internal movement	China	November 2019-February 2020	days	Simulation using stochastic branching processes	yes	No effect
[33]	Hospitalizations, deaths	yes	Stay at home	US	March-April 2020	days	Difference-in-Differences Regression with (weighted) impact dummies	yes	Mitigating effect
[34]	Rt, cases, deaths	na, no	17 NPIs	7 European countries, 114 subnational entities	1 August 2020-9 January 2021	days	Hierarchical Bayesian transmission model	yes	Mitigating effect of workplace closures, strict restrictions on gatherings, school closures
[35]	infections, deaths	yes	Schools closure, workplace closure, cancel public events, stay at home, restrictions on interstate movement	US	21 January-1 May 2020	days	Mixed effects regression	yes	Mitigating effect for social distancing, not for restrictions on internal movement
[36]	deaths	yes	OxCGRT	130 countries	January-May 2020	days	POLS only with time FE	yes	Mitigating effect of earlier and stricter school closure, and workplace closure
[37]	infections	yes	Shutdown	China	January-February 2020	days	Generalized linear model	yes	Mitigating effect of public transport closure, closing entertainment venues and banning public gatherings.

References

1. Askitas N, Tatsiramos K, and Verheyden B. Estimating worldwide effects of non-pharmaceutical interventions on COVID-19 incidence and population mobility patterns using a multiple-event study. *Scientific Reports* 2021; **11**:1972
2. Auger KA et al. Association between statewide school closure and COVID-19 incidence and mortality in the US. *JAMA* 2020; **324**:859–70
3. Banholzer N et al. Impact of non-pharmaceutical interventions on documented cases of COVID-19. *medRxiv* 2020
4. Bendavid E, Oh C, Bhattacharya J, and Ioannidis JPA. Assessing mandatory stay-at-home and business closure effects on the spread of COVID-19. *European journal of clinical investigation* 2021; **51**:e13484
5. Brauner JM et al. Inferring the effectiveness of government interventions against COVID-19. *Science* 2021; **371**
6. Callaway B and Li T. Policy evaluation during a pandemic. *arXiv* 2021. Available from: `http://arxiv.org/pdf/2105.06927v1`
7. Carraro A, Ferrone L, and Squarcina M. Are COVID-19 containment measures equally effective in different world regions? 2020. Available from: `https://EconPapers.repec.org/RePEc:frz:wpaper:wp2020_11.rdf`
8. Chen X and Qiu Z. Scenario analysis of non-pharmaceutical interventions on global COVID-19 transmissions. *arXiv* 2020. Available from: `http://arxiv.org/pdf/2004.04529v2`
9. Chernozhukov V, Kasahara H, and Schrimpf P. Causal impact of masks, policies, behavior on early covid-19 pandemic in the U.S. *Journal of Econometrics* 2021; **220**:23–62
10. Chinazzi M et al. The effect of travel restrictions on the spread of the 2019 novel coronavirus (COVID-19) outbreak. *Science* 2020; **368**:395–400
11. Chu DK et al. Physical distancing, face masks, and eye protection to prevent person-to-person transmission of SARS-CoV-2 and COVID-19: a systematic review and meta-analysis. *The Lancet* 2020; **395**:1973–87

12. Cowling BJ et al. Impact assessment of non-pharmaceutical interventions against coronavirus disease 2019 and influenza in Hong Kong: an observational study. *The Lancet Public Health* 2020; **5**:e279–e288

13. Figueiredo AM de, Codina AD, Figueiredo D, Saez M, and León AC. Impact of lockdown on COVID-19 incidence and mortality in China: an interrupted time series study. *Bulletin of the World Health Organization* 2020; **001**

14. Dehning J et al. Inferring change points in the spread of COVID-19 reveals the effectiveness of interventions. *Science* 2020; **369**

15. Dergiades T, Milas C, and Panagiotidis T. Effectiveness of government policies in response to the COVID-19 outbreak. *SSRN* 2020. Available from: `https://ssrn.com/abstract=3602004`

16. Flaxman S et al. Estimating the effects of non-pharmaceutical interventions on COVID-19 in Europe. *Nature* 2020; **584**:257–61

17. Gatto M et al. Spread and dynamics of the COVID-19 epidemic in Italy: Effects of emergency containment measures. *Proceedings of the National Academy of Sciences of the United States of America* 2020; **117**:10484–91

18. Haug N et al. Ranking the effectiveness of worldwide COVID-19 government interventions. *Nature Human Behaviour* 2020; **4**:1303–12

19. Hong SH, Hwang H, and Park MH. Effect of COVID-19 non-pharmaceutical interventions and the implications for human rights. *International Journal of Environmental Research and Public Health* 2020; **18**

20. Hsiang S et al. The effect of large-scale anti-contagion policies on the COVID-19 pandemic. *Nature* 2020; **584**:262–7

21. Islam N et al. Physical distancing interventions and incidence of coronavirus disease 2019: natural experiment in 149 countries. *BMJ (Clinical research ed.)* 2020; **370**:m2743

22. Kosfeld R, Mitze T, Rode J, and Wälde K. The Covid–19 containment effects of public health measures A spatial difference–in–differences approach. *Journal of Regional Science* 2021; **OnlineFirst**

23. Kraemer MUG et al. The effect of human mobility and control measures on the COVID-19 epidemic in China. *Science* 2020; **368**:493–7

24. Lai S et al. Effect of non-pharmaceutical interventions to contain COVID-19 in China. *Nature* 2020; **585**:410–3

25. Leffler CT, Ing E, Lykins JD, Hogan MC, McKeown CA, and Grzybowski A. Association of country-wide coronavirus mortality with demographics, testing, lockdowns, and public wearing of masks. *The American Journal of Tropical Medicine and Hygiene* 2020; **103**:2400–11

26. Li Y et al. The temporal association of introducing and lifting non-pharmaceutical interventions with the time-varying reproduction number (R) of SARS-CoV-2: a modelling study across 131 countries. *The Lancet Infectious Diseases* 2021; **21**:193–202

27. Liu Y, Morgenstern C, Kelly J, Lowe R, and Jit M. The impact of non-pharmaceutical interventions on SARS-CoV-2 transmission across 130 countries and territories. *BMC medicine* 2021; **19**:40

28. Lyu W and Wehby GL. Community use of face masks and COVID-19: Evidence from a natural experiment of state mandates in the US. *Health affairs (Project Hope)* 2020; **39**:1419–25

29. Maier BF and Brockmann D. Effective containment explains subexponential growth in recent confirmed COVID-19 cases in China. *Science* 2020; **368**:742–6

30. Mitze T, Kosfeld R, Rode J, and Wälde K. Face masks considerably reduce COVID-19 cases in Germany. *Proceedings of the National Academy of Sciences of the United States of America* 2020; **117**:32293–301

31. Orea L and Álvarez IC. How effective has been the Spanish lockdown to battle COVID-19? A spatial analysis of the coronavirus propagation across provinces. 2020. Available from: `https://ideas.repec.org/p/fda/fdaddt/2020-03.html`

32. Quilty BJ et al. The effect of travel restrictions on the geographical spread of COVID-19 between large cities in China: a modelling study. *BMC medicine* 2020; **18**:259

33. Sears J, Villas-Boas JM, Villas-Boas V, and Villas-Boas SB. Are we #Stayinghome to flatten the curve? *medRxiv* 2020. Available from: `https://www.medrxiv.org/content/10.1101/2020.05.23.20111211v3`

34. Sharma M et al. Understanding the effectiveness of government interventions in Europe's second wave of COVID-19. *medRxiv* 2021. Available from: https://www.medrxiv.org/content/10.1101/2021.03.25.21254330v1

35. Siedner MJ et al. Social distancing to slow the US COVID-19 epidemic: Longitudinal pretest-posttest comparison group study. *PLoS Medicine* 2020; **17**:e1003244

36. Stokes J, Turner AJ, Anselmi L, Morciano M, and Hone T. The relative effects of non-pharmaceutical interventions on early Covid-19 mortality: natural experiment in 130 countries. *medRxiv* 2020. Available from: https://www.medrxiv.org/content/10.1101/2020.10.05.20206888v1

37. Tian H et al. An investigation of transmission control measures during the first 50 days of the COVID-19 epidemic in China. *Science* 2020; **368**:638–42
